# The impact of metabolic endotoxaemia on the browning process in human adipocytes

**DOI:** 10.1186/s12916-023-02857-z

**Published:** 2023-04-19

**Authors:** Farah Omran, Alice M. Murphy, Awais Z. Younis, Ioannis Kyrou, Jana Vrbikova, Vojtech Hainer, Petra Sramkova, Martin Fried, Graham Ball, Gyanendra Tripathi, Sudhesh Kumar, Philip G. McTernan, Mark Christian

**Affiliations:** 1 Division of Biomedical Sciences, Warwick Medical School, University of Warwick, Coventry, CV2 2DX UK; 2 Department of Biosciences, School of Science and Technology, Nottingham Trent University, Nottingham, NG11 8NS UK; 3 Warwickshire Institute for the Study of Diabetes, Endocrinology and Metabolism (WISDEM), University Hospitals Coventry and Warwickshire NHS Trust, Coventry, CV2 2DX UK; 4 Centre for Sport, Exercise and Life Sciences, Research Institute for Health & Wellbeing, Coventry University, Coventry, CV1 5FB UK; 5 Aston Medical School, College of Health and Life Sciences, Aston University, Birmingham, B4 7ET UK; 6 Warwick Medical School, University of Warwick, Coventry, CV4 7AL UK; 7 Institute of Endocrinology, Prague, Czech Republic; 8 OB Clinic, Prague, Czech Republic; 9 Medical Technology Research Centre, Anglia Ruskin University, Cambridge, UK; 10 Human Sciences Research Centre, College of Life and Natural Sciences, University of Derby, Derby, DE22 1GB UK

**Keywords:** Obesity, Endotoxin, Adipocyte browning, Mitochondria, Lipopolysaccharide, Human adipocytes, Bariatric surgery

## Abstract

**Background:**

Dysfunctional adipose tissue (AT) is known to contribute to the pathophysiology of metabolic disease, including type 2 diabetes mellitus (T2DM). This dysfunction may occur, in part, as a consequence of gut-derived endotoxaemia inducing changes in adipocyte mitochondrial function and reducing the proportion of BRITE (brown-in-white) adipocytes. Therefore, the present study investigated whether endotoxin (lipopolysaccharide; LPS) directly contributes to impaired human adipocyte mitochondrial function and browning in human adipocytes, and the relevant impact of obesity status pre and post bariatric surgery.

**Methods:**

Human differentiated abdominal subcutaneous (AbdSc) adipocytes from participants with obesity and normal-weight participants were treated with endotoxin to assess in vitro changes in mitochondrial function and BRITE phenotype. Ex vivo human AbdSc AT from different groups of participants (normal-weight, obesity, pre- and 6 months post-bariatric surgery) were assessed for similar analyses including circulating endotoxin levels.

**Results:**

Ex vivo AT analysis (lean & obese, weight loss post-bariatric surgery) identified that systemic endotoxin negatively correlated with BAT gene expression (*p* < 0.05). In vitro endotoxin treatment of AbdSc adipocytes (lean & obese) reduced mitochondrial dynamics (74.6% reduction; *p* < 0.0001), biogenesis (81.2% reduction; *p* < 0.0001) and the BRITE phenotype (93.8% reduction; *p* < 0.0001). Lean AbdSc adipocytes were more responsive to adrenergic signalling than obese AbdSc adipocytes; although endotoxin mitigated this response (92.6% reduction; *p* < 0.0001).

**Conclusions:**

Taken together, these data suggest that systemic gut-derived endotoxaemia contributes to both individual adipocyte dysfunction and reduced browning capacity of the adipocyte cell population, exacerbating metabolic consequences. As bariatric surgery reduces endotoxin levels and is associated with improving adipocyte functionality, this may provide further evidence regarding the metabolic benefits of such surgical interventions.

**Supplementary Information:**

The online version contains supplementary material available at 10.1186/s12916-023-02857-z.

## Background

Low-grade chronic systemic inflammation, characterized by increased circulating pro-inflammatory factors, is strongly associated with obesity and obesity-related diseases, such as type 2 diabetes mellitus (T2DM) and cardiovascular disease (CVD) [1, 2]. Indeed, this chronic inflammation constitutes a major risk factor for cardio-metabolic morbidity and mortality [2, 3]. Adipose tissue (AT) directly produces and releases several pro-inflammatory adipokines/cytokines that contribute to the development of this chronic inflammation and the associated diseases[1]. In addition, obesity is known to impair the permeability of the gut wall, resulting in a ‘leaky gut’ which enables gut-derived inflammatory agents (*e.g.* endotoxin, also referred to as lipopolysaccharide; LPS) to enter the bloodstream [4, 5]. Endotoxin forms part of the outer membrane of gram-negative bacteria and, once in the circulation, can elicit a marked inflammatory response in adipocytes, as our previous work has shown [6–10]. Overall, the combination of chronic inflammation, dietary factors (*e.g.* a high fat diet) and excess weight gain in obesity can promote adipocyte dysfunction [11].

Obesity is associated with the alteration of the proportion of both white (WAT) and brown adipose tissue (BAT) in the body, with a subsequent metabolic impact [12–15]. WAT stores excess energy as triglycerides (TGs), however with excess and sustained weight gain the volume of WAT expands to a point where TGs spill over into ectopic sites (*e.g.* in the liver, skeletal muscle and pancreas), inducing insulin resistance and metabolic dysfunction [16]. In contrast, BAT, which is rich in mitochondria, can dissipate energy as heat via non-shivering thermogenesis, which contributes to the removal of plasma TGs, mitigates ectopic lipid storage, and improves insulin sensitivity and glucose uptake [16–20]. In obesity, BAT mass/activity appears to reduce, with multiple studies indicating that individuals with obesity have reduced BAT compared with their lean counterparts [18, 21, 22]. Of note, it is possible for WAT to possess adipocytes with a brown phenotype known as BRITE (brown-in-white) or beige adipocytes, following a browning process in response to cold adaptation or other stimuli. Differences in susceptibility to browning between WAT from lean individuals and individuals with obesity have not yet been investigated; however, weight loss via interventions such as bariatric surgery has been shown to enhance the browning of adipocytes [23–27]. Although chronic inflammation has a profound effect on the metabolic function of adipocytes, the underlying mechanism(s) mediating this browning effect remains unclear.

To date, there is a paucity of data on the direct influence of endotoxin on adipocyte browning and the associated impact on the production and health of mitochondria [28, 29]. BAT is known to have a large number of well-developed mitochondria, and therefore mitochondrial health and biogenesis are indicators that BRITE adipocytes are functioning correctly. This includes constant fusion and fission of mitochondria to maintain their shape, distribution and size in order to function optimally and carry out quality control processes [30, 31]. Transcriptional regulation plays an important role in managing mitochondrial biogenesis and dynamics, with key proteins involved in these processes, which are highly regulated at the transcriptional level [32–34]. As such, studying mitochondrial biogenesis and dynamics at the transcriptional level in response to endotoxin is expected to provide novel insight into the impact on BRITE cells and their mitochondrial health.

Therefore, this study sought to investigate the direct impact of endotoxin on both adipocyte browning and mitochondrial health, utilizing both in vitro and in vivo human data from lean individuals and individuals with obesity, as well as individuals with obesity-related T2DM who have undergone bariatric surgery. Accordingly, the aims of the present study were to investigate: (1) the relationship between endotoxin and adipocyte browning in human WAT in vivo*;* (2) the impact of endotoxin on the transcriptional characteristics of the BRITE phenotype in vitro; (3) the effect of endotoxin on mitochondrial genes in BRITE adipocytes in vitro*;* and (4) the potential of inflammation as a mediator of LPS action within WAT.

## Methods

### Ethics and study design

Human WAT samples were obtained from participants with (i) body mass index (BMI): 18.5–24.9 kg/m^2^, (lean: *n* = 44), (ii) BMI: 25–29.9 kg/m^2^ (overweight: *n* = 49); and (iii) BMI: > 30 kg/m^2^ (obese: *n* = 63). Of the 63 participants with obesity, 26 were Caucasian women with severe obesity (BMI > 35 kg/m^2^) and T2DM who underwent bariatric surgery at the OB clinic, Prague, Czech Republic; either biliopancreatic diversion (BPD: *n* = 8), laparoscopic greater curvature plication (LGCP; *n* = 10), or laparoscopic adjustable gastric banding (LAGB; *n* = 8). For this study group, eligibility criteria was BMI > 35 kg/m^2^ with or without comorbidities. WAT samples and fasted blood samples were collected both pre- and 6-months post-surgery at the Institute of Endocrinology, Prague, Czech Republic. All other study participants were non-diabetic, pre-menopausal Caucasian women who underwent non-emergency abdominal surgeries at University Hospital Coventry and Warwickshire (UHCW) NHS Trust, Coventry, UK (2013–2018). Exclusion criteria for the present study included diseases, such as cancer and Cushing’s syndrome, as well as medications, such as glucocorticoids, incretin mimetics, insulin, and thiazolidinediones which could interfere with the objectives of the study and were considered potential confounders. AT biopsies from abdominal subcutaneous (Sc) and omental (Om) fat depots were obtained, alongside fasted serum samples. Ethical approval was obtained from the Local Research Ethics Committees, and all study participants provided written and informed consent in accordance with the Declaration of Helsinki.

### Blood biochemistry and anthropometry

Biochemical and anthropometric measurements were performed at the time of surgery, as well as 6 months post-surgery for those undergoing bariatric surgery. Participants underwent a 10-h overnight fast, after which venous blood was collected in chilled EDTA-containing tubes with and without aprotinin (for glucose and insulin measurements). Accordingly, serum samples were prepared, aliquoted and frozen at -80 °C until assayed. Serum glucose, HbA1c and lipids were determined using the Cobas 6000 analyzer. Insulin resistance was assessed using the homeostatic model assessment of insulin resistance (HOMA-IR) according to the following equation: HOMA-IR = fasting glucose (mmol/L) x fasting insulin (mIU/L)/22.5, as previously described [35]. The Friedwald formula [36] was used to compute serum levels of LDL cholesterol. Body weight was measured to the nearest 0.5 kg and height to the nearest 1 cm. For those undergoing bariatric surgery, percentage of excess weight loss was calculated according to the following equation: (preoperative weight – postoperative weight) / (preoperative weight – ideal body weight) × 100, and body fat mass was measured using the bioimpedance method (Tanita TBF-300; Tanita corporation).

### Primary human pre-adipocyte isolation

Abdominal Sc AT was digested with collagenase to isolate pre-adipocyte cells, as previously described [37]. Briefly, AT was incubated with collagenase class 1 (Worthington, UK) for 30 min before being filtered through a cotton mesh and centrifuged. The pellet was then re-suspended in Dulbecco’s modified Eagle’s medium with high glucose (DMEM/F12) containing 10% FBS and 10 µg/mL transferrin. Cells were then cultured at 37 °C, 5% CO_2_.

### Primary human pre-adipocyte differentiation and treatment

Once confluent, cells were grown for a further two days before being incubated in differentiation media (DMEM/F12, 3% FBS and Differentiation Supplement Mix (Promocell, Heidelberg, Germany)) for four days. Differentiation was induced in the presence or absence of 2 µM rosiglitazone (Rosi, #71,740, Cayman Chemical, Cambridge, UK) to promote browning, 100 ng/mL lipopolysaccharide (LPS, 100 ng/mL, E. Coli O55:B5, #L6529, Sigma-Aldrich, UK) to induce inflammation, or a combination of the two. Following this, cells were maintained in nutrition media (DMEM/F12 with Nutrition Supplement Mix (Promocell)) for 10 days at which point they were fully differentiated. Cells were then incubated in basal media (DMEM/F12 with 0.5% BSA) for 24 h before being harvested, or treated with 10 µM isoproterenol (Iso, #I6504, Sigma-Aldrich, UK) for 5 h to stimulate an adrenergic response and induce uncoupling protein 1 (UCP1) expression before harvesting.

### RNA isolation and quantification

For adipose tissue, 100 mg of frozen tissue was homogenized in TRI Reagent® (#T9424 Sigma-Aldrich, UK); for cell culture, cells were lysed in TRI Reagent. Total RNA was then extracted with chloroform (#J67241.AP, VWR International Ltd., UK) (0.2 mL, TRI reagent®:Chloroform 5:1 v/v) and isopropanol (0.5 mL, TRI reagent®: Isopropanol 2:1 v/v). Samples were measured on a spectrophotometer at 260 nm.

### cDNA synthesis and qRT-PCR

Samples were digested with DNase I (DNase I kit, #AMP-D1 Sigma-Aldrich, UK) and cDNA synthesis was undertaken using a Sigma-Aldrich mRNA reverse transcription kit (#M1302-40KU, UK) according to the manufacturer’s instructions. Primers for mRNA were synthesized by Sigma Aldrich (see Additional file 1, Table S1). All assays were carried out in duplicate using SYBR Green mastermix (#S4438-500RXN Sigma-Aldrich, UK), using L19 as a housekeeping control gene.

### Protein determination and western blot analysis

Cultured primary adipocytes (*n* = 3) were lysed in radioimmunoprecipitation assay buffer (#9806, Cell Signalling, US) supplemented with protease and phosphatase inhibitor cocktail (#11,836,153,001, Roche, Switzerland). Once harvested, protein concentration was determined via Bradford Assay (#5,000,006, Biorad, US). Western blotting was performed as described previously [38]. In brief, 30ug of protein was loaded onto a denaturing polyacrylamide gel and transferred on to polyvinylidene difluoride membranes (#IPVH00010, Millipore, US). Membranes were blocked in a 5% bovine serum albumin (BSA) solution, incubated with antibodies against UCP1 (1:2000, #ab10983, Abcam, UK) and subsequently with secondary antibodies conjugated to horse radish peroxidase. Equal protein loading was confirmed using antibodies against β-Actin (1:1000, Cell Signalling, US). Proteins were visualised using the XBOX chemi-luminescence imaging system (Syngene, US) and band intensities were quantified with Fiji software [39] (ImageJ, US).

### Oxygen Consumption Rate (OCR) measurements

A Seahorse XFe24 Extracellular Flux Analyser (Seahorse Bioscience, Santa Clara, CA, US) was used to measure OCR as described elsewhere [40]. Primary pre-adipocytes were seeded on to 24-well Seahorse Mitcroplates coated with 0.1% gelatine, and were differentiated and treated as detailed above. Media was changed to Seahorse XF media one hour before undertaking the assays. The XFe Cell Mito Stress Test was carried out using 2 μM Oligomycin, 2 μM FCCP and 0.5 μM rotenone/antimycin (*n* = 5); preliminary experiments were used to determine optimal drug concentrations (data not shown). In a separate assay, isoproterenol was injected into the wells containing differentiated, treated primary adipocytes at a final concentration of 10 μM to determine their ability to respond to an adrenergic stimulus (*n* = 5). Values from both assays were normalised to total protein.

### Endotoxin serum measurements

Serum endotoxin was measured using the EndoLISA assay (Hyglos, Germany) as per manufacturer’s instructions. Briefly, samples were added to wells pre-coated with endotoxin- specific phage binding protein before being washed. An assay reagent was then added which generated a fluorescent compound in the presence of endotoxin. Fluorescence was then measured against a standard curve to determine endotoxin concentration.

### Artificial neural network inference

BAT and inflammatory genes were selected based on their association with phenotype from an Artificial Neural Network (ANN)-based data-mining step. The ANN model was validated using Monte Carlo cross-validation to minimize the risk of over-fitting and to optimize the generality of the model, as described previously [41]. This resulted in a phenotype enriched molecule set which was investigated further within an Artificial Neural Network Inference (ANNi) algorithm [41], including analysis of BMI and endotoxin. Within this ANN inference a matrix of interactions was generated based on signal directions and prediction weights. This matrix was then collapsed by taking the sum of values to and from each gene to determine the most influential and most influenced features. The interactions from this matrix were also presented in a cytoscape map [42].

### Statistical analysis

Statistical analyses were performed using the SPSS 21.0 software [43] and GraphPad Prism 7.04. Data were examined for normality according to the D'Agostino & Pearson normality test. Visual inspection of the data histograms, normal Q-Qplots and box plots were examined, with skewness and kurtosis z-values accepted at (-1.96—1.96). Analysis of the varying BMI cohort (*n* = 136) was performed via one-way ANOVA (if parametric) or Kruskal–Wallis test (if non-parametric) followed by Tukey's (if parametric) or Dunn's (if non-parametric) multiple comparisons test to define significant differences between individual groups. Differences between pre- and post-surgery time-points in the bariatric surgery cohort (*n* = 26) were assessed via paired two-tailed T-Test (if parametric) and the Wilcoxon signed ranks test (if non-parametric). For Pearson correlation analyses, data were log-transformed prior to analysis, if non-parametric. Two-way ANOVA, followed by Tukey’s multiple comparison test, was performed for all comparisons between different cell culture treatments. *P*-values of < 0.05 were considered statistically significant. Unless otherwise specified, data are reported as mean ± standard error of the mean (SEM) with statistical differences compared to control indicated with *p < 0.05, **p < 0.01, ***p < 0.001, ****p < 0.0001.

## Results

### Anthropometric and metabolic variables for study participants

Pertinent clinical, anthropometric and biochemical data for the study groups (Table 1) were stratified based on BMI, (lean (*n* = 44), overweight (*n* = 49) and obesity (*n* = 63)) and evaluated. Participants who were overweight or obese were compared with lean participants. Analysis of these cases indicated that fasting insulin levels were significantly increased in both the overweight and obese group (both *p*-values < 0.05). Additionally, fasting glucose (*p* < 0.0001) and LDL (*p* < 0.05) levels were significantly increased in the obese group. The latter obese group also exhibited significantly lower fasting HDL levels than lean participants (*p* < 0.001).

**Table 1:** Clinical, anthropometric, and fasting biochemical characteristics of participants based on BMI and bariatric surgery status

	**Lean**	**Overweight**	**Obese**	**Pre-Surgery**	**Post-Surgery**
n	44	49	63	26	26
Age (years)	32.07 ± 1.2	31.56 ± 1.4	39.93 ± 1.7***^####^	54.50 ± 3.41	54.50 ± 3.41
BMI (Kg/m^2^)	22.14 ± 0.7	27.30 ± 0.8****	36.35 ± 1.0****^####^	42.21 ± 3.24	36.70 ± 3.14^++++^
Glucose (mmol/l)	3.58 ± 0.1	3.63 ± 0.1	5.92 ± 0.4****^####^	9.22 ± 1.57	6.96 ± 0.83^++++^
LDL (mmol/l)	4.56 ± 0.2	4.46 ± 0.2	3.82 ± 0.2**^#^	3.08 ± 0.54	2.67 ± 0.40^+^
HDL (mmol/l)	1.74 ± 0.1	1.53 ± 0.1	1.35 ± 0.1****	1.08 ± 0.16	1.04 ± 0.18
TGs (mmol/l)	3.11 ± 0.2	3.26 ± 0.1	2.65 ± 0.2^#^	1.83 ± 0.65	1.39 ± 0.40^+^
Insulin (pmol/l)	39.79 ± 4.3	64.58 ± 6.3*	62.97 ± 5.9*	31.90 ± 11.26	17.16 ± 5.49^++++^

Selected pertinent clinical, anthropometric and fasting biochemical characteristics of the study participants based on body mass index (BMI) and bariatric surgery status; participants with (i) BMI: 18.5–24.9 kg/m^2^, (lean: *n* = 44); (ii) BMI: 25–29.9 kg/m^2^ (overweight: *n* = 49); and (iii) BMI: > 30 kg/m^2^ (obese: *n* = 63). Data are presented as mean ± SEM. All samples were taken in a fasted state. One-way ANOVA or the Kruskal–Wallis test were used to test significance between group comparisons; *p < 0.05, **p < 0.01, ***p < 0.001, ****p < 0.0001 for lean vs. overweight and lean vs. obese, ^#^ *p* < 0.05, ^##^ *p* < 0.01, ^###^ *p* < 0.001, ^####^ *p* < 0.0001 for overweight vs. obese. Significant differences between measurements taken pre- and 6 months post-bariatric surgery were assessed using two-tailed paired T-Test or Wilcoxon signed ranks test; ^+^*p* < 0.05, ^++^*p* < 0.01, ^+++^*p* < 0.001, ^++++^*p* < 0.0001

*BMI* Body mass index, *TGs* Triglycerides, *LDL* Low-density lipoprotein, *HDL* High-density lipoprotein

### Change in endotoxin post-bariatric surgery correlates with BAT gene expression

**Fig. 1:**
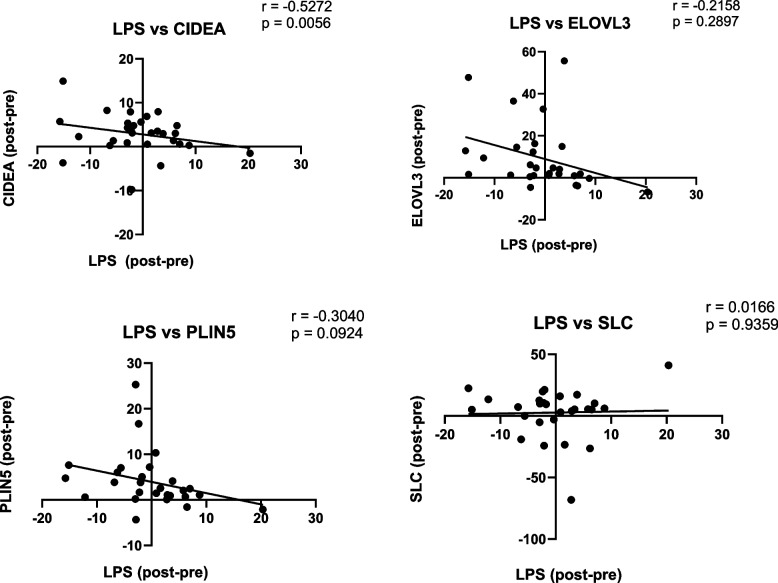
Circulating Endotoxin Correlates with BAT gene expression changes pre- and 6 months post-bariatric surgery. Correlations between the changes in endotoxin (LPS) and brown adipose tissue (BAT) genes (**A**) CIDEA (cell death activator CIDE-A), **B** ELOVL3 (elongation of very long chain fatty acids protein 3), **C** PLIN5 (perilipin 5), and (**D**) SLC27A2 (solute carrier family 27 member 2) following bariatric surgery. Change was measured by subtracting the pre- value from the post- value; *r* values were calculated using the Pearson correlation test and *p* < 0.05 was considered significant

Paired serum and AT samples were collected from study participants undergoing bariatric surgery and analyzed for circulating endotoxin (LPS) and AT brown gene expression both pre- and 6 months post-surgery. Brown genes CIDEA (cell death activator CIDE-A), ELOVL3 (elongation of very long-chain fatty acids protein 3), PLIN5 (perilipin 5) and SLC27A2 (solute carrier family 27 member 2) were included in this analysis. Our findings show that, compared with pre-surgery levels, circulating endotoxin significantly decreased post-surgery, whilst brown gene expression increased, resulting in a negative correlation between endotoxin and ELOVL3, PLIN5 and CIDEA (Fig. 1, *p* < 0.05). Artificial neural network inference analysis revealed that SLC27A2 was most influenced by inflammatory genes, with its expression pre-surgery negatively affected by CD14, IL6, IL10 and CD68. Pre-surgery CD14 expression also influenced SLC27A2 levels post-surgery (see Additional file 1, Fig. S1).

### BMI correlates with reduced BAT gene expression

**Fig. 2:**
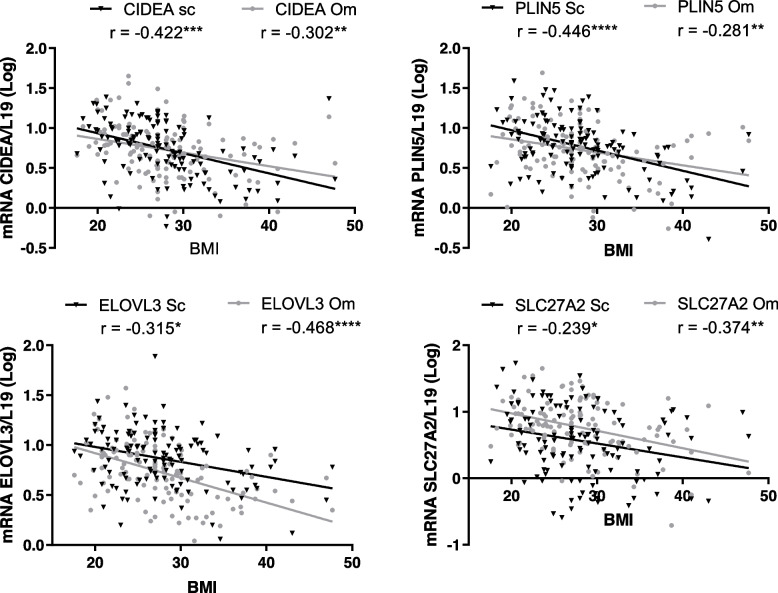
Body mass index (BMI) Correlates with Reduced Brown Adipose Tissue Genes. The expression of key brown fat genes Cell death-inducing DFFA-like effector A (CIDEA), ELOVL fatty acid elongase 3 (ELOVL3), perilipin 5 (PLIN5) and Solute Carrier Family 27 Member 2 (SLC27A2) was measured in subcutaneous (Sc) and omental (Om) adipose tissue depots via RT-PCR using L19 as a housekeeping control. Pearson correlation test was used to calculate *r* values; all genes had a strong negative correlation with BMI. *p < 0.05, **p < 0.01, ***p < 0.001, ****p < 0.0001

To establish the relationship between BMI and the BRITE genotype, the expression levels of key brown fat genes including CIDEA, ELOVL3, PLIN5 and SLC27A2 were analyzed in both subcutaneous (Sc) and omental (Om) AT samples from patients with a range of BMIs. A strong negative correlation between BMI and BAT genes was observed with a maximum *r* value of -0.422 for CIDEA in Sc, and -0.468 for ELOVL3 in Om (Fig. 2, *p* < 0.05). UCP1 gene expression was low in both Sc and Om AT samples with no significant change between lean and obese, or Sc and Om depots (see Additional file 1, Fig. S2).

### Inflammation is increased in abdominal AT samples with BMI

To determine if inflammation was increased with increasing BMI as expected, pro-inflammatory markers IL6, tumour necrosis factor alpha (TNFα), MCP1 and interleukin 1 beta (IL1β) were measured in the lean, overweight and obese study groups in both Sc and Om AT depots. All these pro-inflammatory markers were significantly upregulated in participants with obesity compared with lean individuals (see Additional file 1, Fig. S3, *p* < 0.05), with an observed maximum threefold difference for IL1β (see Additional file 1, Fig. S3D2, *p* < 0.001).

### Inflammatory genes correlate with reduced BAT genes

As expression of both BAT and inflammatory genes were shown to significantly change with increasing BMI, the direct relationship between the two was also investigated, and a Pearson correlation test was carried out to determine any significant correlations. This analysis highlighted that there was a strong negative correlation between the BAT and inflammatory genes in Om AT, with a less apparent negative correlation present in Sc AT (Table 2). Utilizing the artificial neural network inference (ANNi algorithm), a network was created demonstrating the interactions between inflammatory markers and brown fat genes in Sc (see Additional file 1, Fig. S4) and Om (see Additional file 1, Fig. S5). This network graph revealed that in Sc, CIDEA and PLIN5 were the most negatively influenced brown genes and IL6, PLIN5 and ELOVL3 had the most influence on other genes. In Om, SLC27A2 was the most influenced brown gene, whilst CIDEA had the highest level of influence on others, followed by IL6 and PLIN5.

**Table 2:** Correlations between studied pro-inflammatory and brown adipose tissue (BAT) related genes

		**IL6**		**MCP1**		**TNFα**		**IL1β**	
		**Pearson’s r**	***P* value**	**Pearson’s r**	***P* value**	**Pearson’s r**	***P* value**	**Pearson’s r**	***P* value**
**CIDEA**	**Sc**	-0.01	0.91	0.01	0.93	-0.05	0.55	0.03	0.78
	**Om**	-0.33***	**2.00E-04**	-0.31***	**6.00E-04**	-0.12	0.21	-0.40****	**7.25E-06**
**ELOVL3**	**Sc**	-0.23**	**0.01**	-0.16	0.08	-0.06	0.52	-0.13	0.17
	**Om**	-0.33***	**2.00E-04**	-0.30***	**7.00E-04**	-0.05	0.62	-0.35***	**1.00E-04**
**PLIN5**	**Sc**	-0.20*	**0.03**	-0.05	0.58	-0.26**	**3.00E-03**	-0.16	0.08
	**Om**	-0.26**	**3.00E-03**	-0.22*	**0.02**	-0.17	0.05	-0.23*	**0.01**
**SLC27A2**	**Sc**	0.07	0.47	0.004	0.96	-0.10	0.28	0.10	0.28
	**Om**	-0.13	0.19	-0.12	0.19	0.01	0.91	-0.29**	**1.00E-03**

The correlation of genes for interleukin-6 (IL6), monocyte chemotactic protein-1 (MCP1), tumour necrosis factor-alpha (TNFα), and interleukin-1beta (IL1β) with BAT-related genes, *i.e.* cell death-inducing DFFA-like effector A (CIDEA), ELOVL fatty acid elongase 3 (ELOVL3), perilipin 5 (PLIN5) and solute carrier family 27 member 2 (SLC27A2), was assessed using the Pearson correlation test. Significant correlations are highlighted in bold font, *p < 0.05, **p < 0.01, ***p < 0.001, ****p < 0.0001

### Endotoxin reduced adipocyte browning in primary human adipocytes

**Fig. 3:**
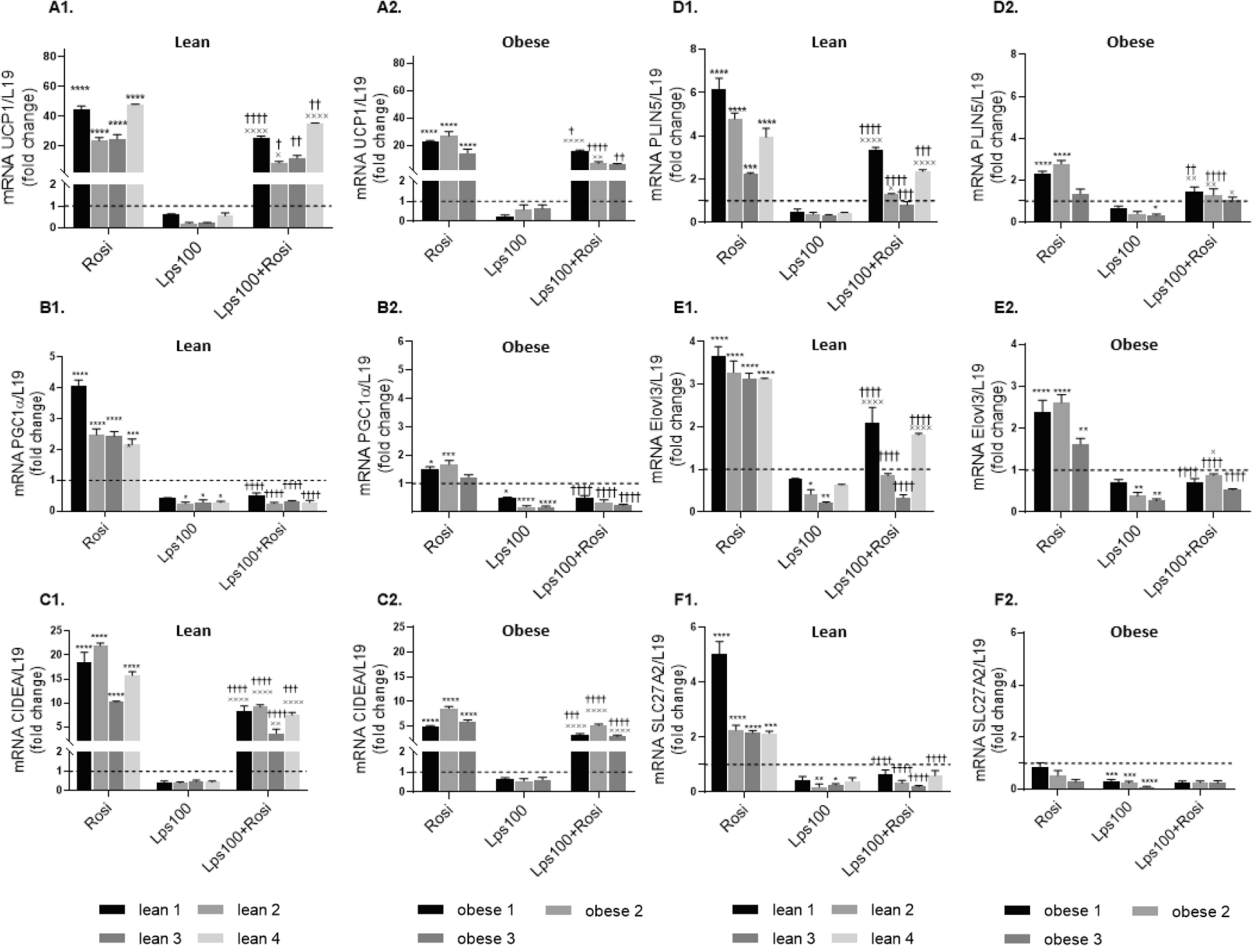
Impact of Endotoxin (LPS) on Human Primary Adipocyte Browning. Lean (A1/B1/C1/D1/E1/F1) and obese (A2/B2/C2/D2/E2/F2) primary human adipocytes were differentiated with/without 2 µM rosiglitazone (Rosi), 100 ng/mL lipopolysaccharide (LPS100) or a combination of the two. Browning genes uncoupling protein 1 (UCP1), peroxisome proliferator-activated receptor gamma coactivator 1-alpha (PGC1α), cell death-inducing DFFA-like effector A (CIDEA), ELOVL fatty acid elongase 3 (ELOVL3), perilipin 5 (PLIN5) and Solute Carrier Family 27 Member 2 (SLC27A2) were analyzed using qRT-PCR with L19 as a housekeeping control. Data represent mean ± standard error of the mean (SEM). The two-way ANOVA test was used to test significance levels; *p < 0.05, **p < 0.01, ***p < 0.001, ****p < 0.0001 compared to control; † *p* < 0.05, †† *p* < 0.01, ††† *p* < 0.001, †††† *p* < 0.0001 compared to Rosi treatment; × *p* < 0.05, ×  ×  ×  × *p* < 0.0001 compared to LPS treatment

To investigate the direct impact of endotoxin on BAT gene expression, lean and obese primary human adipocytes were grown and differentiated with or without a gut-derived endotoxin fragment (LPS, 100 ng/mL) and/or 2 µM rosiglitazone, which replicates the brown phenotype. The gene expression of adipocyte protein 2 (aP2) was measured, indicating that treatments did not impact differentiation (see Additional file 1, Fig. S6). Differentiation with endotoxin reduced the expression of BAT genes UCP1, PGC1α, CIDEA, PLIN5, ELOVL3 and SLC27A2 compared to control. Differentiation with rosiglitazone significantly upregulated most BAT genes, with a maximum upregulation of 22-fold (*p* < 0.001); however, upregulation occurred to a much greater extent in adipocytes from lean individuals compared to obese. This upregulation indicates that lean adipocytes may be more susceptible to browning than obese adipocytes. The upregulation of BAT genes was significantly reduced when endotoxin was included in the treatment (*p* < 0.001), suggesting that endotoxin and therefore obesity-related endotoxaemia may impair the browning process (Fig. 3).

### Endotoxin reduces the responsiveness of BRITE cells to adrenergic stimuli

**Fig. 4:**
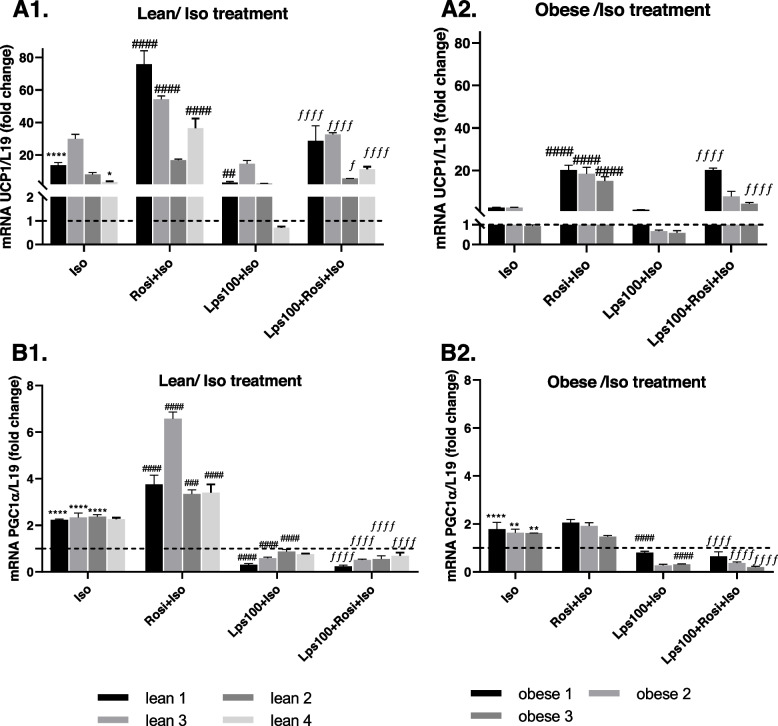
Impact of Endotoxin (LPS) on BRITE Adipocyte Response to Adrenergic Stimulus. Lean (**A1, B1**) and obese (**A2, B2**) primary human adipocytes were differentiated with or without 2 µM rosiglitazone (Rosi), 100 ng/mL lipopolysaccharide (LPS100) or a combination of the two. When fully differentiated, cells were treated with 10 µM isoproterenol (Iso). BAT genes uncoupling protein 1 (UCP1) and peroxisome proliferator-activated receptor gamma coactivator 1-alpha (PGC1α) were analyzed using RT-PCR with L19 as a housekeeping control. Data are presented as mean ± SEM. Two-way ANOVA was used to test significance; *p < 0.05, **p < 0.01, ***p < 0.001, ****p < 0.0001 compared to control; # *p* < 0.05, ## *p* < 0.01, ### *p* < 0.001, #### *p* < 0.0001 compared to Iso treatment; ƒ *p* < 0.05, ƒƒ *p* < 0.01, ƒƒƒ *p* < 0.001, ƒƒƒƒ *p* < 0.0001 compared to Rosi + Iso treatment

Following the observation that endotoxin reduces the level of browning of adipocytes, we investigated whether endotoxin also impacts the ability of BRITE cells to respond to an adrenergic stimulus. Lean and obese primary human adipocytes were grown and differentiated with 2 µM rosiglitazone, endotoxin (LPS, 100 ng/mL), or a combination of the two. Once differentiated, cells were treated with or without 10 µM isoproterenol. Isoproterenol stimulates an adrenergic response similar to cold exposure in brown adipocytes, which should induce UCP1 and PGC1α expression if the cells are functioning correctly. As presented in Fig. 4, isoproterenol treatment significantly increased the expression of UCP1 and PGC1α in most cases, both with and without rosiglitazone treatment (*p* < 0.0001). However, adipocytes from lean individuals experienced a much higher increase than those from individuals with obesity. Endotoxin treatment significantly reduced the expression of both genes in the presence and absence of rosiglitazone (*p* < 0.0001), indicating that endotoxin reduces the capacity of these cells to respond to an adrenergic stimulus (Fig. 4).

### Endotoxin impairs mitochondrial function

**Fig. 5:**
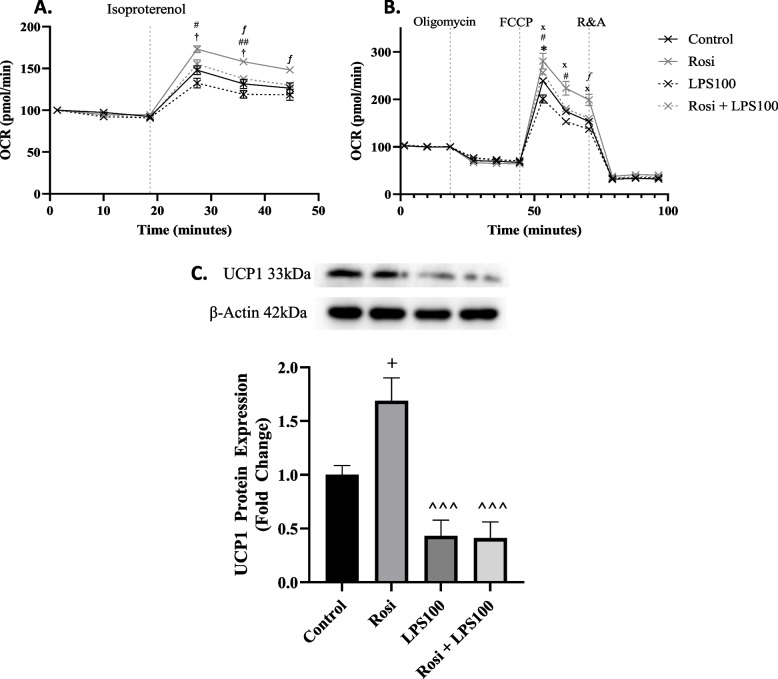
Impact of Endotoxin (LPS) on Mitochondrial Function. Primary human adipocytes were differentiated in the presence of 2 µM rosiglitazone (Rosi), 100 ng/mL lipopolysaccharide (LPS) or a combination of the two. **A** Following differentiation, isoproterenol was injected (to a final concentration of 10 µM) whilst oxygen consumption rate (OCR) was measured. **B** A Seahorse Mito Stress Test was also performed on the differentiated cells to assess key parameters of mitochondrial respiration. Dotted lines indicate injections into media of the specific compounds: isoproterenol, oligomycin, carbonyl cyanite-4 (trifluoromethoxy) phenylhydrazone (FCCP) and rotenone/antimycin A (R&A). **C** UCP1 protein expression was assessed in differentiated cells via Western blot. One-way ANOVA was carried out to assess significant differences. Seahorse data: * *p* < 0.05 control vs. LPS; # *p* < 0.05, ## *p* < 0.01 LPS vs. Rosi; *x p* < 0.05 LPS vs. Rosi + LPS; *f* Rosi vs. Rosi + LPS; † control vs. Rosi. UCP1 data: + *p* < 0.05 compared to control; ^^^ *p* < 0.001 compared to Rosi

To explore the impact of endotoxin on the adrenergic response further, a Seahorse analyser was used to measure oxygen consumption rate (OCR) whilst isoproterenol was injected into the media of lean primary adipocytes treated with 2 µM rosiglitazone, endotoxin (LPS, 100 ng/mL), or a combination of the two. An increase in OCR represents the adrenergic response. Cells treated with rosiglitazone exhibited an 83% increase with isoproterenol which was significantly different to the 60% increase in control cells, and 46% increase in cells treated with endotoxin (*p* < 0.05). Cells treated with both rosiglitazone and endotoxin had an increased OCR of 72% following isoproterenol injection, however this was not significantly different to cells treated with rosiglitazone alone (Fig. 5A). A mitochondrial stress test was also carried out, indicating that maximal respiration was increased with rosiglitazone treatment compared to control cells (*p* < 0.05), whilst endotoxin significantly decreased the maximal respiration (*p* < 0.05, Fig. 5B). Previous studies suggest that these differences may be due to UCP1 [44]. As such, UCP1 protein expression was assessed. Rosiglitazone caused a 1.7-fold increase in UCP1 expression (*p* < 0.05), whilst the inclusion of endotoxin with the rosiglitazone treatment caused a 59% decrease in expression compared to control (*p* < 0.001). Endotoxin on its own had a similar impact, reducing UCP1 expression by 57% (*p* < 0.01, Fig. 5C).

### Endotoxin reduces mitochondrial dynamics

**Fig. 6:**
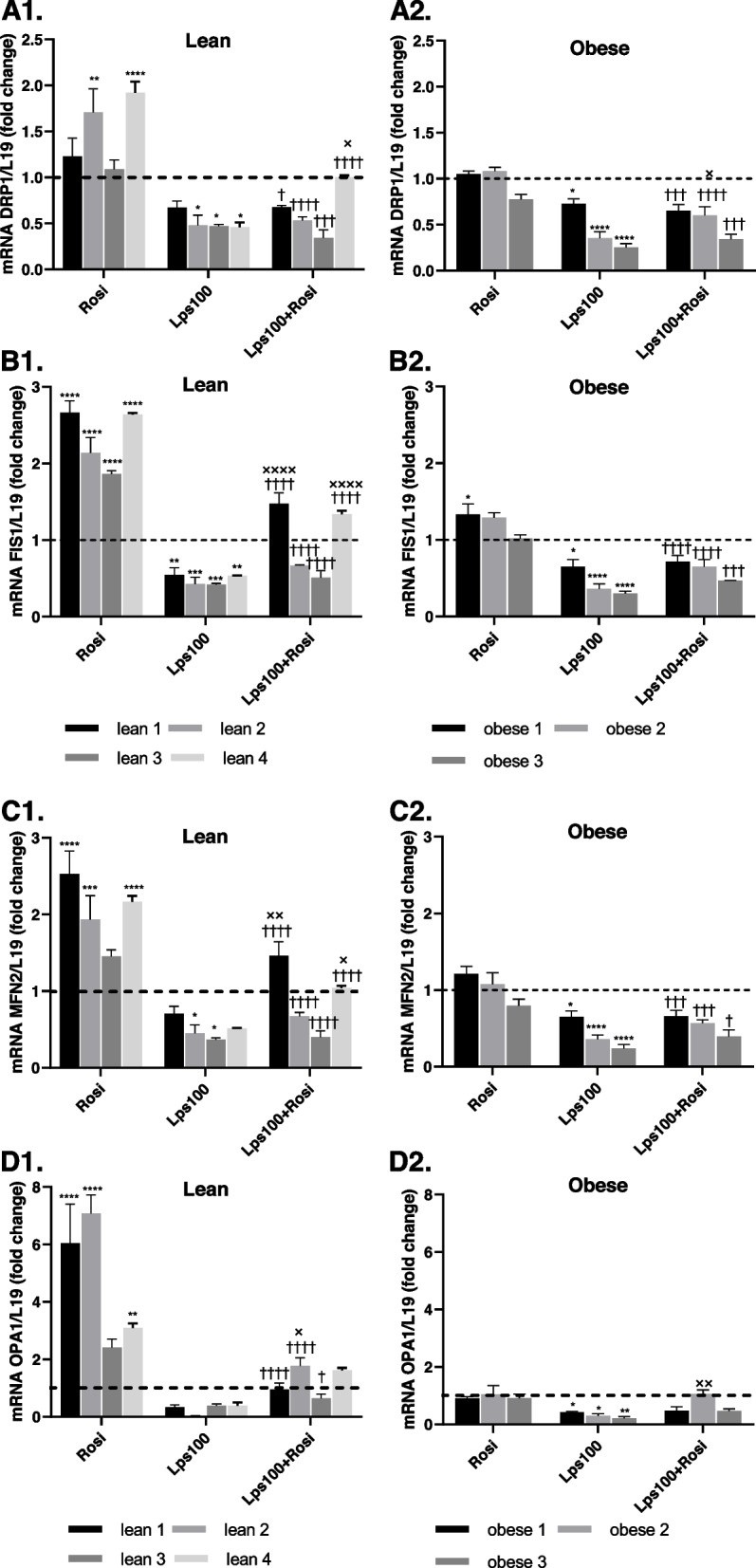
Impact of Endotoxin (LPS) on Mitochondrial Dynamics. Following differentiation of lean (**A1, B1, C1, D1**, *n* = 4) and obese (**A2, B2, C2, D2**, *n* = 3) primary human adipocytes with 2 µM rosiglitazone (Rosi), 100 ng/mL lipopolysaccharide (LPS100) or a combination of the two, mitochondrial fission genes dynamin-related protein 1 (DRP1) and mitochondrial fission 1 (FIS1), as well as fusion genes mitofusin 2 (MFN2) and mitochondrial dynamin like GTPase (OPA1) were analyzed via RT-PCR, with L19 as a housekeeping control. Data represent mean ± SEM. The two-way ANOVA test was used to test significance; *p < 0.05, **p < 0.01, ***p < 0.001, ****p < 0.0001 compared to control, † *p* < 0.05, †† *p* < 0.01, ††† *p* < 0.001, †††† *p* < 0.0001 compared to Rosi treatment, x *p* < 0.05, xx *p* < 0.01, xxx *p* < 0.001, xxxx*p* < 0.0001 compared to LPS

To further explore the impact of endotoxin on BRITE adipocyte function, mitochondrial health was investigated via their ability to undergo fission and fusion following endotoxin treatment. Fission genes dynamin-related protein 1 (DRP1) and mitochondrial fission 1 (FIS1), as well as fusion genes mitofusin 2 (MFN2) and mitochondrial dynamin like GTPase (OPA1) were analyzed in lean and obese primary human adipocytes following treatment with rosiglitazone, endotoxin (LPS) or a combination of the two. Adipocytes from lean individuals had increased expression of fission and fusion genes when treated with rosiglitazone, whereas adipocytes from individuals with obesity experienced very little change with rosiglitazone treatment (Fig. 6). Differentiating cells in the presence of endotoxin significantly reduced the expression of all mitochondrial dynamic genes in both the presence and absence of rosiglitazone (*p* < 0.001), suggesting that endotoxin reduces the ability of the adipocytes to maintain healthy mitochondria (Fig. 6).

### Endotoxin reduces mitochondrial biogenesis

**Fig. 7:**
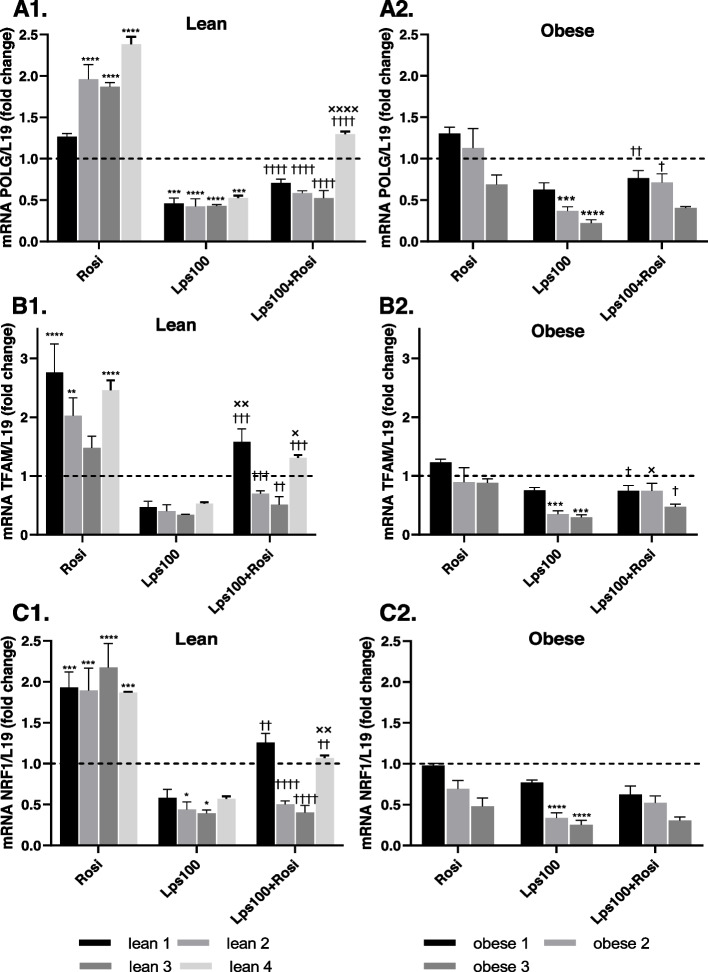
The Impact of Endotoxin (LPS) on Mitochondrial Biogenesis. Primary human adipocytes were differentiated with or without rosiglitazone (Rosi), 100 ng/mL lipopolysaccharide (LPS100) or a combination of the two. Mitochondrial biogenesis genes DNA polymerase gamma (POLG), nuclear respiratory factor 1 (NRF1) and mitochondrial transcription factor A (TFAM) were measured using RT-PCR, with L19 as a housekeeping control. Data represent mean ± SEM. The two-way ANOVA test was used to test significance; *p < 0.05, **p < 0.01, ***p < 0.001, ****p < 0.0001 compared to control, † *p* < 0.05, †† *p* < 0.01, ††† *p* < 0.001, †††† *p* < 0.0001 compared to Rosi treatment, x *p* < 0.05, xx *p* < 0.01, xxx *p* < 0.001, xxxx *p* < 0.0001 compared to LPS

Mitochondrial biogenesis genes citrate synthase (CS), DNA polymerase gamma (POLG), nuclear respiratory factor 1 (NRF1) and mitochondrial transcription factor A (TFAM) were analyzed via RT-PCR following the treatment of primary human adipocytes with rosiglitazone, endotoxin (LPS) or a combination of the two. Lean adipocytes experienced a maximum 2.8-fold increase (*p* < 0.001) in mitochondrial biogenesis when treated with rosiglitazone, compared with obese adipocytes which did not significantly differ from control. Endotoxin treatment significantly reduced expression of all mitochondrial biogenesis genes both in the presence and absence of rosiglitazone, suggesting that endotoxin negatively impacts the production of new mitochondria in adipocytes (Fig. 7).

### Endotoxin induces inflammation in primary human adipocytes

To investigate inflammation as a potential mechanism mediating the effect of endotoxin on adipocyte browning, both lean and obese primary human adipocytes were treated with endotoxin (LPS, 100 ng/mL), 2 µM rosiglitazone, or a combination of the two. Following endotoxin treatment, genes for the pro-inflammatory factors interleukin 6 (IL6) and monocyte chemotactic protein-1 (MCP1) were significantly upregulated. A maximum 15-fold increase in lean adipocytes, and a maximum tenfold increase in obese adipocytes (see Additional file 1, Fig. S7, *p* < 0.001) was observed. This suggests that endotoxin induces an inflammatory response in lean white adipocytes that appears blunted in obese white adipocytes. In addition, IL6 and MCP1 were significantly upregulated by a maximum of eightfold by endotoxin in rosiglitazone treated cells, indicating that endotoxin also induces inflammation in BRITE adipocytes.

## Discussion

These studies highlight for the first time the damaging influence gut derived endotoxin has on human adipocyte function through inhibiting the browning process and reducing mitochondrial health, as well as providing insight as to why obesity itself exacerbates the inflammatory response. Specifically, these human studies have shown that endotoxin prevents the induction of adipocyte browning in primary white adipocytes; whilst differentiated BRITE adipocytes have reduced responsiveness to adrenergic stimuli when treated with endotoxin, particularly noted in cells cultured from obese subjects. In vivo human AT analysis indicated that endotoxin negatively impacts the expression of the BRITE phenotype in obesity and highlighted the associated benefit of bariatric surgery on reducing endotoxin levels and increasing WAT browning. In vitro analysis revealed that endotoxin treatment impaired mitochondrial respiration, dynamics and biogenesis in BRITE adipocytes with BAT genes also being influenced by inflammatory genes, highlighting the downstream influence endotoxin has on inflammation. As such, these findings suggest that endotoxin contributes to obesity-associated disorders by impairing adipocyte browning and mitochondrial health via inflammatory pathways.

In vivo assessment of the relationship between endotoxin and adipocyte browning was investigated by exploring in patient correlations pre- and post-bariatric surgery, as well as modelling analysis with an artificial neural network inference. This revealed that endotoxin was negatively correlated with brown fat genes in WAT, and BAT-related genes were directly negatively influenced by inflammatory genes. This highlights the potential for increased fat storage to impact on WAT browning capacity via inflammation. Accordingly, our studies monitored the expression of BAT-related genes in both lean and obese human primary adipocytes in vitro when exposed to endotoxin alongside the browning agent, rosiglitazone. Endotoxin consistently reduced the expression of BAT-related genes in both lean and obese adipocytes, indicating that less browning occurs in adipocytes following exposure to endotoxin. This suggests that individuals with increased circulating levels of endotoxin, such as those noted in individuals who are overweight or have obesity, may have a reduced ability to produce BRITE adipocytes. With impaired capacity for fatty acid oxidation due to depleted BRITE adipocytes, this may lead to increased ectopic lipid accumulation and subsequent insulin resistance and related comorbidities [45]. In addition, rosiglitazone promoted the expression of BAT-related genes in lean adipocytes to a much higher level than in obese adipocytes. As such, adipocytes from individuals with obesity may be less susceptible to a browning stimulus than those from lean individuals. A possible reason for this could be the increased level of inflammation in obesity, which has been shown to impair adipocyte browning [46]. Additionally, fibroblast growth factor 21 (FGF21), a key mediator of fatty acid oxidation and lipid metabolism, which has been demonstrated to enhance WAT browning, is reduced in obesity [47]. GLP-1 and β3-adrenergic receptor agonists, such as Liraglutide and Mirabegron, result in BAT activation and have been shown to reduce fat mass [48, 49]. The findings we report have implications in the search for potential obesity treatments, since agents that are shown to induce browning in lean individuals may not have the same capacity in individuals with obesity. Importantly, browning of AT and stimulation of BAT induced by bariatric surgery and/or drugs, may counteract a reduction in metabolic rate frequently associated with energy restriction and weight loss [50].

Moreover, to determine the impact of endotoxin on non-shivering thermogenesis of BRITE adipocytes, the responsiveness of adipocytes to adrenergic stimulation following exposure to endotoxin was monitored. Differentiating with endotoxin consistently reduced the adrenergic response of both lean and obese adipocytes, indicating that endotoxin impairs the ability of BRITE adipocytes to induce non-shivering thermogenesis at the transcription level when required. As a result, increased levels of endotoxin are likely to reduce the metabolic rate, as energy is stored as lipids instead of being dissipated through heat, further promoting obesity and metabolic dysfunction. To our knowledge, this is the first study showing such results in human primary adipocytes, which are in line with previous studies in mice [28, 51–53]. Furthermore, adipocytes from lean individuals were more responsive to the adrenergic stimulus without endotoxin than those from individuals with obesity. This is consistent with our previous findings, as well as the relevant literature, which indicates that individuals with obesity have blunted non-shivering thermogenesis response under the same cold stimulation compared with lean individuals [54]. This reduced responsiveness to induce non-shivering thermogenesis may, at least partly, be linked to the endotoxaemia present in obesity, as our results indicate that endotoxin impairs the adrenergic response.

Since non-shivering thermogenesis in brown and BRITE adipocytes relies on functional mitochondria, our studies investigated the impact of endotoxin on the function, biogenesis and dynamics of mitochondria, which is indicative of their health. Rosiglitazone increased maximal respiration of the mitochondria whilst endotoxin impaired such increase, indicating that endotoxin negatively impacts the ability of mitochondria to cope with a physiological energy demand. Rosiglitazone also upregulated biogenesis genes, as well as fission and fusion genes, in lean individuals, but had no effect on those with obesity. Alongside results on BAT-related gene expression, this indicates that, whilst rosiglitazone induces some browning in adipocytes from individuals with obesity, these adipocytes do not have the same increase in mitochondrial biogenesis and dynamics as those in adipocytes from lean individuals. This provides further insight as to why adipocytes from individuals with obesity were observed to have a reduced adrenergic response, since studies suggest that thermogenesis is regulated by mitochondrial dynamics in brown adipocytes [45, 55]. Furthermore, endotoxin treatment impaired the increase in mitochondrial biogenesis and dynamics genes in response to rosiglitazone in lean adipocytes, to the point that they were displaying a more obese genotype. This reduction in both mitochondrial biogenesis and dynamics means that quality control is impaired, and damaged mitochondria are not able to be replaced at an adequate rate. Similarly, the mitochondria have a diminished ability to adapt to cellular stresses and metabolic demands [33]. As such mitochondrial dysfunction occurs, which has been proposed as a cause of AT inflammation and is known to contribute to the risk of developing obesity-related comorbidities [56]. 

To investigate endotoxin as a mediator of inflammation, in vitro studies assessed the impact of endotoxin on lean and obese primary human adipocytes. As it is known that circulating endotoxin levels increase with BMI [57], this study assessed pro-inflammatory markers in vivo in lean, overweight and obese cohorts. As expected, the expression of pro-inflammatory markers was increased in overweight and obese groups compared with lean, in both Sc and Om AT, which is consistent with previous studies [58]. Furthermore, a strong negative correlation was observed between BMI and BAT-related genes in both Sc and Om AT. These findings are consistent with the existing relevant literature and highlight the possibility that pro-inflammatory genes may be associated with BAT-related gene expression [12, 13]. Whilst these correlations have been shown before, this is the first study to follow up with an investigation into the relationship between BAT and inflammatory genes. Indeed, further investigation revealed strong negative correlations between BAT and inflammatory genes in Om AT, with weaker negative correlations in Sc AT. This is possibly due to the higher levels of inflammation noted in Om compared to Sc AT [59, 60]. ANN analysis provided further insight, highlighting the direct relationship between inflammatory and BAT genes, indicating that inflammation as a downstream factor of endotoxin can also prevent the browning of adipocytes. Whilst we have considered IL6 as a pro-inflammatory marker, there is also evidence to suggest it can influence adipocyte browning [61], however the results observed with IL6 in this study were in line with the other pro-inflammatory markers assessed.

Our study is limited by only including female participants, who are at lower risk of developing metabolic disease than males until menopause. Future studies should include males in order to explore the effects of metabolic endotoxemia on adipocyte browning in men *vs* women. A longitudinal study of participants who did not undergo bariatric surgery would be preferred in order to allow each subject to act as their own control, however this was not possible.

## Conclusions

Based on these findings, it is proposed that endotoxin may prevent adipocyte browning and impair mitochondrial respiration, biogenesis and dynamics in human adipocytes, thus contributing to obesity-related metabolic dysfunction, including dyslipidaemia and ectopic AT deposition in T2DM. Targeting endotoxin may therefore be a viable option to prevent the development of obesity-related cardio-metabolic diseases by enhancing mitochondrial health and efficiency in adipocytes, whilst also increasing the number of BRITE adipocytes and improving their function.

## Supplementary Information

**Additional file 1: Table S1.** Primer sequences. Sequences of primers used during qRT-PCR. **Figure S1.** Artificial neural network inference of brown and inflammatory genes pre- and post-bariatric surgery. A network map showing the strength and direction of interactions between genes measured before and after bariatric surgery. **Figure S2.** UCP1 expression in lean and obese subcutaneous and omental adipose tissue. Gene expression of UCP1 in subcutaneous and omental adipose tissue from lean participants and participants with obesity. **Figure S3.** Inflammatory markers are increased with BMI. Bar graphs showing the expression of inflammatory genes across participants divided into lean, overweight and obese cohorts. **Figure S4. **Artificial neural network inference of brown and inflammatory genes in subcutaneous adipose tissue. A network map showing the strength and direction of interactions between genes in subcutaneous adipose tissue. **Figure S5.** Artificial neural network inference of brown and inflammatory genes in omental adipose tissue. A network map showing the strength and direction of interactions between genes in omental adipose tissue. **Figure S6.** AP2 gene expression during differentiation with LPS treatment. Gene expression of AP2 demonstrating that differentiation of adipocytes is not affected by cellular treatments. **Figure S7.** Effect of LPS on inflammation in primary human adipocytes. Expression of inflammatory genes in primary human adipocytes following LPS treatment.

**Additional file 2. **

## Data Availability

The datasets used and/or analysed during the current study are available from the corresponding author on reasonable request.

## Abbreviations

AbdSC: Abdominal subcutaneous
ANN: Artificial neural network
ANNi: Artificial neural network inference
AT: Adipose tissue
BAT: Brown adipose tissue
BMI: Body mass index
BPD: Biliopancreatic diversion
BRITE: Brown-in-white
BSA: Bovine serum albumin
CD14: Cluster of differentiation 14
CD68: Cluster of differentiation 68
CIDEA: Cell death activator CIDE-A
CS: Citrate synthase
CVD: Cardiovascular disease
DMEM/F12: Dulbecco’s Modified Eagle Medium/Nutrient Mixture F12
DRP1: Dynamin-related protein 1
ELOVL3: Elongation of very long-chain fatty acids protein 3
FBS: Foetal bovine serum
FGF21: Fibroblast growth factor 21
FIS1: Mitochondrial fission 1
GLP1: Glucagon-like peptide 1
HDL: High density lipoprotein
HOMA-IR: Homeostatic Model Assessment for Insulin Resistance
IL10: Interleukin 10
IL1β: Interleukin 1 beta
IL6: Interleukin 6
LAGB: Laparoscopic adjustable gastric banding
LDL: Low density lipoprotein
LGCP: Laparoscopic greater curvature plication
LPS: Lipopolysaccharide
MCP1: Monocyte chemoattractant protein-1
MFN2: Mitofusin 2
NRF1: Nuclear respiratory factor 1
OM: Omental
OPA1: Mitochondrial dynamin like GTPase
PGC1α: Peroxisome proliferator-activated receptor-gamma coactivator-1 alpha
PLIN5: Perilipin 5
POLG: DNA polymerase subunit gamma
SC: Subcutaneous
SLC27A2: Solute carrier family 27 member 2
T2DM: Type 2 diabetes mellitus
TFAM: Mitochondrial transcription factor A
TGs: Triglycerides
TNFα: Tumour necrosis factor alpha
UCP1: Uncoupling protein 1
UHCW: University Hospitals Coventry and Warwickshire
WAT: White adipose tissue

## References

[1] Burhans MS, Hagman DK, Kuzma JN, Schmidt KA, Kratz M (2019). Contribution of adipose tissue inflammation to the development of type 2 diabetes mellitus. Compr Physiol.

[2] Zatterale F, Longo M, Naderi J, Raciti GA, Desiderio A, Miele C (2020). Chronic Adipose Tissue Inflammation Linking Obesity to Insulin Resistance and Type 2 Diabetes. Front Physiol.

[3] Furman D, Campisi J, Verdin E, Carrera-Bastos P, Targ S, Franceschi C (2019). Chronic inflammation in the etiology of disease across the life span. Nat Med.

[4] Fasano A (2017). Gut permeability, obesity, and metabolic disorders: Who is the chicken and who is the egg?. Am J Clin Nutr.

[5] Piya MK, Harte AL, McTernan PG (2013). Metabolic endotoxaemia: Is it more than just a gut feeling?. Curr Opin Lipidol.

[6] Varma MC, Kusminski CM, Azharian S, Gilardini L, Kumar S, Invitti C, et al. Metabolic endotoxaemia in childhood obesity. BMC Obes. 2016;3:1-8. 10.1186/s40608-016-0083-7 PMC4728817 26819711

[7] Creely SJ, McTernan PG, Kusminski CM, Fisher FM, da Silva NF, Khanolkar M, et al. Lipopolysaccharide activates an innate immune system response in human adipose tissue in obesity and type 2 diabetes. Am J Physiol Endocrinol Metab. 2007;292:E740–7. 10.1152/ajpendo.00302.2006 17090751

[8] Harte AL, da Silva NF, Creely SJ, McGee KC, Billyard T, Youssef-Elabd EM, et al. Elevated endotoxin levels in non-alcoholic fatty liver disease. J Inflamm. 2010;7:1-10. 10.1186/1476-9255-7-15 PMC2873499 20353583

[9] Harte AL, Varma MC, Tripathi G, McGee KC, Al-Daghri NM, Al-Attas OS (2012). High fat intake leads to acute postprandial exposure to circulating endotoxin in type 2 diabetic subjects. Diabetes Care.

[10] Martinez de la Escalera L, Kyrou I, Vrbikova J, Hainer V, Sramkova P, Fried M, et al. Impact of gut hormone FGF-19 on type-2 diabetes and mitochondrial recovery in a prospective study of obese diabetic women undergoing bariatric surgery. BMC Med. 2017;15:1-9. 10.1186/s12916-017-0797-5 PMC5311731 28202005

[11] Bowser SM, Mcmillan RP, Boutagy NE, Tarpey MD, Smithson AT, Osterberg KL (2020). Serum endotoxin, gut permeability and skeletal muscle metabolic adaptations following a short term high fat diet in humans. Metabolism.

[12] Nascimento EBM, Sparks LM, Divoux A, van Gisbergen MW, Broeders EPM, Jörgensen JA (2018). Genetic Markers of Brown Adipose Tissue Identity and In Vitro Brown Adipose Tissue Activity in Humans. Obesity.

[13] Al-Amrani A, AbdelKarim M, AlZabin M, Alzoghaibi M (2019). Low expression of brown and beige fat genes in subcutaneous tissues in obese patients. Arch Med Sci.

[14] Pellegrinelli V, Carobbio S, Vidal-Puig A (2016). Adipose tissue plasticity: how fat depots respond differently to pathophysiological cues. Diabetologia.

[15] Orava J, Nuutila P, Noponen T, Parkkola R, Viljanen T, Enerbäck S (2013). Blunted metabolic responses to cold and insulin stimulation in brown adipose tissue of obese humans. Obesity.

[16] Chait A, den Hartigh LJ (2020). Adipose Tissue Distribution, Inflammation and Its Metabolic Consequences, Including Diabetes and Cardiovascular Disease. Front Cardiovasc Med.

[17] Bond LM, Ntambi JM (2018). UCP1 deficiency increases adipose tissue monounsaturated fatty acid synthesis and trafficking to the liver. J Lipid Res.

[18] Matsushita M, Yoneshiro T, Aita S, Kameya T, Sugie H, Saito M (2014). Impact of brown adipose tissue on body fatness and glucose metabolism in healthy humans. Int J Obes (Lond).

[19] Koksharova E, Ustyuzhanin D, Philippov Y, Mayorov A, Shestakova M, Shariya M (2017). The Relationship Between Brown Adipose Tissue Content in Supraclavicular Fat Depots and Insulin Sensitivity in Patients with Type 2 Diabetes Mellitus and Prediabetes. Diabetes Technol Ther.

[20] Chondronikola M, Volpi E, Børsheim E, Porter C, Annamalai P, Enerbäck S (2014). Brown adipose tissue improves whole-body glucose homeostasis and insulin sensitivity in humans. Diabetes.

[21] Leitner BP, Huang S, Brychta RJ, Duckworth CJ, Baskin AS, McGehee S (2017). Mapping of human brown adipose tissue in lean and obese young men. Proc Natl Acad Sci U S A.

[22] Vijgen GHEJ, Bouvy ND, Teule GJJ, Brans B, Schrauwen P, van Marken Lichtenbelt WD. Brown adipose tissue in morbidly obese subjects. PLoS One. 2011;6:e17247. 10.1371/journal.pone.0017247 PMC3044745 21390318

[23] Dadson P, Hannukainen JC, Din MU, Lahesmaa M, Kalliokoski KK, Iozzo P (2018). Brown adipose tissue lipid metabolism in morbid obesity: Effect of bariatric surgery-induced weight loss. Diabetes Obes Metab.

[24] Vijgen GHEJ, Bouvy ND, Teule GJJ, Brans B, Hoeks J, Schrauwen P (2012). Increase in Brown Adipose Tissue Activity after Weight Loss in Morbidly Obese Subjects. J Clin Endocrinol Metab.

[25] Hankir MK, Seyfried F (2020). Do Bariatric Surgeries Enhance Brown/Beige Adipose Tissue Thermogenesis?. Front Endocrinol.

[26] Adami GF, Carbone F, Montecucco F, Camerini G, Cordera R (2019). Adipose Tissue Composition in Obesity and After Bariatric Surgery. Obes Surg.

[27] Chen Y, Yang J, Nie X, Song Z, Gu Y (2018). Effects of Bariatric Surgery on Change of Brown Adipocyte Tissue and Energy Metabolism in Obese Mice. Obes Surg.

[28] Okla M, Wang W, Kang I, Pashaj A, Carr T, Chung S (2015). Activation of Toll-like receptor 4 (TLR4) attenuates adaptive thermogenesis via endoplasmic reticulum stress. J Biol Chem.

[29] Gavaldà-Navarro A, Moreno-Navarrete JM, Quesada-López T, Cairó M, Giralt M, Fernández-Real JM (2016). Lipopolysaccharide-binding protein is a negative regulator of adipose tissue browning in mice and humans. Diabetologia.

[30] Mahdaviani K, Benador IY, Su S, Gharakhanian RA, Stiles L, Trudeau KM (2017). Mfn2 deletion in brown adipose tissue protects from insulin resistance and impairs thermogenesis. EMBO Rep.

[31] Tilokani L, Nagashima S, Paupe V, Prudent J (2018). Mitochondrial dynamics: Overview of molecular mechanisms. Essays Biochem.

[32] Jornayvaz FR, Shulman GI (2010). Regulation of mitochondrial biogenesis. Essays Biochem.

[33] Ferree A, Shirihai O (2012). Mitochondrial dynamics: The intersection of form and function. Adv Exp Med Biol.

[34] Pearce SF, Rebelo-Guiomar P, D’Souza AR, Powell CA, Van Haute L, Minczuk M (2017). Regulation of Mammalian Mitochondrial Gene Expression: Recent Advances. Trends Biochem Sci.

[35] Matthews DR, Hosker JP, Rudenski AS, Naylor BA, Treacher DF, Turner RC (1985). Homeostasis model assessment: insulin resistance and β-cell function from fasting plasma glucose and insulin concentrations in man. Diabetologia.

[36] Friedewald WT, Levy RI, Fredrickson DS (1972). Estimation of the Concentration of Low-Density Lipoprotein Cholesterol in Plasma, Without Use of the Preparative Ultracentrifuge. Clin Chem.

[37] McTernan PG, Anwar A, Eggo MC, Barnett AH, Stewart PM, Kumar S (2000). Gender differences in the regulation of P450 aromatase expression and activity in human adipose tissue. Int J Obes.

[38] Kusminski CM, Da Silva NF, Creely SJ, Fisher FM, Harte AL, Baker AR (2007). The in vitro effects of resistin on the innate immune signaling pathway in isolated human subcutaneous adipocytes. J Clin Endocrinol Metab.

[39] Schindelin J, Arganda-Carreras I, Frise E, Kaynig V, Longair M, Pietzsch T (2012). Fiji: an open-source platform for biological-image analysis. Nat Methods.

[40] Jackisch L, Murphy AM, Kumar S, Randeva H, Tripathi G, McTernan PG (2020). Tunicamycin-Induced Endoplasmic Reticulum Stress Mediates Mitochondrial Dysfunction in Human Adipocytes. J Clin Endocrinol Metab.

[41] Tong DL, Boocock DJ, Dhondalay GKR, Lemetre C, Ball GR (2014). Artificial Neural Network Inference (ANNI): A Study on Gene-Gene Interaction for Biomarkers in Childhood Sarcomas. PLoS ONE.

[42] Shannon P, Markiel A, Ozier O, Baliga NS, Wang JT, Ramage D (2003). Cytoscape: a software environment for integrated models of biomolecular interaction networks. Genome Res.

[43] IBM Corp (2012). Released 2012. IBM SPSS Statistics for Windows, Version 21.0.

[44] Yeo CR, Agrawal M, Hoon S, Shabbir A, Shrivastava MK, Huang S (2017). SGBS cells as a model of human adipocyte browning: A comprehensive comparative study with primary human white subcutaneous adipocytes. Sci Rep.

[45] Pisani DF, Barquissau V, Chambard JC, Beuzelin D, Ghandour RA, Giroud M (2018). Mitochondrial fission is associated with UCP1 activity in human brite/beige adipocytes. Mol Metab.

[46] Villarroya F, Cereijo R, Gavaldà-Navarro A, Villarroya J, Giralt M (2018). Inflammation of brown/beige adipose tissues in obesity and metabolic disease. J Intern Med.

[47] Cui X-B, Chen S-Y. White adipose tissue browning and obesity. 2017. 10.7555/JBR.31.20160101. 10.7555/JBR.31.20160101 PMC5274505 28808180

[48] Beiroa D, Imbernon M, Gallego R, Senra A, Herranz D, Villarroya F (2014). GLP-1 agonism stimulates brown adipose tissue thermogenesis and browning through hypothalamic AMPK. Diabetes.

[49] Cero C, Lea HJ, Zhu KY, Shamsi F, Tseng Y-H, Cypess AM. β3-Adrenergic receptors regulate human brown/beige adipocyte lipolysis and thermogenesis. JCI Insight. 2021;6:e139160. 10.1172/jci.insight.139160 PMC8262278 34100382

[50] Melby CL, Paris HL, Foright RM, Peth J. Attenuating the Biologic Drive for Weight Regain Following Weight Loss: Must What Goes Down Always Go Back Up? Nutrients. 2017;9. 10.3390/nu9050468 PMC5452198 28481261

[51] Bae J, Ricciardi CJ, Esposito D, Komarnytsky S, Hu P, Curry BJ (2014). Activation of pattern recognition receptors in brown adipocytes induces inflammation and suppresses uncoupling protein 1 expression and mitochondrial respiration. Am J Physiol Cell Physiol.

[52] Nøhr MK, Bobba N, Richelsen B, Lund S, Pedersen SB. Inflammation downregulates UCP1 expression in brown adipocytes potentially via SIRT1 and DBC1 interaction. Int J Mol Sci. 2017;18. 10.3390/ijms18051006 PMC5454919 28481291

[53] Okla M, Zaher W, Alfayez M, Chung S (2018). Inhibitory Effects of Toll-Like Receptor 4, NLRP3 Inflammasome, and Interleukin-1β on White Adipocyte Browning. Inflammation.

[54] van Marken Lichtenbelt WD, Schrauwen P. Implications of nonshivering thermogenesis for energy balance regulation in humans. Am J Physiol Regul Integr Comp Physiol. 2011;301:R285–96. 10.1152/ajpregu.00652.2010 21490370

[55] Wikstrom JD, Mahdaviani K, Liesa M, Sereda SB, Si Y, Las G (2014). Hormone-induced mitochondrial fission is utilized by brown adipocytes as an amplification pathway for energy expenditure. EMBO J.

[56] Woo CY, Jang JE, Lee SE, Koh EH, Lee KU (2019). Mitochondrial dysfunction in adipocytes as a primary cause of adipose tissue inflammation. Diabetes Metab J.

[57] Radilla-Vázquez RB, Parra-Rojas I, Martínez-Hernández NE, Márquez-Sandoval YF, Illades-Aguiar B, Castro-Alarcón N (2016). Gut Microbiota and Metabolic Endotoxemia in Young Obese Mexican Subjects. Obes Facts.

[58] Creely SJ, McTernan PG, Kusminski CM, Fisher ff. M, Silva NF da, Khanolkar M, et al. Lipopolysaccharide activates an innate immune system response in human adipose tissue in obesity and type 2 diabetes. 2007;292:740–7. 10.1152/ajpendo.00302.2006. 10.1152/ajpendo.00302.2006 17090751

[59] Ziegler AK, Damgaard A, Mackey AL, Schjerling P, Magnusson P, Olesen AT, et al. An anti-inflammatory phenotype in visceral adipose tissue of old lean mice, augmented by exercise. Sci Rep. 2019;9. 10.1038/s41598-019-48587-2 PMC6700172 31427677

[60] Alvehus M, Burén J, Sjöström M, Goedecke J, Olsson T (2010). The human visceral fat depot has a unique inflammatory profile. Obesity.

[61] Kristóf E, Klusóczki Á, Veress R, Shaw A, Combi ZS, Varga K (2019). Interleukin-6 released from differentiating human beige adipocytes improves browning. Exp Cell Res.

