# Supplementary material for: The impact of metabolic endotoxaemia on the browning process in human adipocytes
Source: BMC Med. 2023 Apr 19;21:154. doi: 10.1186/s12916-023-02857-z (PMC10116789; doi:10.1186/s12916-023-02857-z)

**Additional File 1:**

**Table S1 - Primer Sequences**

| **Gene** | **Primer sequence** | | **Product length (bp)** |
| --- | --- | --- | --- |
| *L19* | Forward  Reverse | 5’- GCGGAAGGGTACAGCCAA-3’  5’- GCAGCCGGCGCAAAA-3’ | 77 |
| *Ucp1* | Forward  Reverse | 5’- GTGTGCCCAACTGTGCAATG-3’  5’- CCAGGATCCAAGTCGCAAGA-3’ | 95 |
| *CIDEA* | Forward  Reverse | 5’- CATGTATGAGATGTACTCCGTGTC-3’  5’- GAGTAGGACAGGAACCGCAG-3’ | 90 |
| *PLIN5* | Forward  Reverse | 5’- GTGGATCACTTCCTGCCCAT-3’  5’- CTCCTCTGATCCTCCACCGA-3’ | 95 |
| *ELOVL3* | Forward  Reverse | 5’- AAGGAACGCAAGGGCTTCAA-3’  5’- TGCCCCCAGGATACTGAAGA-3’ | 81 |
| *SLC27A2* | Forward  Reverse | 5’- AAGGCCCCGGTTTCTAAGAA-3’  5’- TTAAAGCCCTCCTCCACCAG-3’ | 90 |
| *aP2* | Forward  Reverse | 5’- TGTTGCAGAAATGGGATGGAAA-3’  5’- CAACGTTCCCTTGGCTTATGCT-3’ | 134 |
| *MCP1* | Forward  Reverse | 5’-CAGCAGCAAGTGTCCCAAAG-3’  5’-GAATCCTGAACCCACTTCTGCTT-3’ | 93 |
| *IL6* | Forward  Reverse | 5’- AGTAGTGAGGAACAAGCCAGA-3’  5’- GTCAGGGGTGGTTATTGCATC-3’ | 102 |
| *TNFα* | Forward  Reverse | 5’- GCTGCACTTTGGAGTGATCG-3’  5’- GTCACTCGGGGTTCGAGAAG-3’ | 109 |
| *IL1β* | Forward  Reverse | 5’- CGCCAGTGAAATGATGGCT-3’  5’- GAGGGCAGAGGTCCAGGTC-3’ | 110 |
| *CS* | Forward  Reverse | 5’- CAGGGTATCAGCCGAACCAA -3’  5’- TTGCTGCAACACAAGGTAGC-3’ | 85 |
| *PGC1α* | Forward  Reverse | 5’- TGAAGAGCGCCGTGTGATT-3’  5’- CAGTTCTGTCCGTGTTGTGTCA-3’ | 61 |
| *Fis1* | Forward  Reverse | 5’- AAGACGTAATCCCGCTGTTCC-3’  5’- TGACATCCGTAAAGGCATCG-3’ | 78 |
| *DRP1* | Forward  Reverse | 5’- TGTCTTCTTCGTAAAAGGTTGCC-3’  5’- ACAAGCATCAGCAAAGTCTGG-3’ | 108 |
| *MFN2* | Forward  Reverse | 5’- GCAGCTTGTCATCAGCTACAC-3’  5’- ATGAGCAAAGGTCCCAGACAG-3’ | 76 |
| *OPA1* | Forward  Reverse | 5’- AGGGAACAGCTCTGAAAGCAT-3’  5’- TCACTTGGTGTGCCTTTAGCA-3’ | 104 |
| *NRF1* | Forward  Reverse | 5’- TGGTCATCTCACCTCCCTGT-3’  5’- GAATGCCAACCACGGTCA-3’ | 102 |
| *TFAM* | Forward  Reverse | 5’- ACCCATATTTAAAGCTCAGAACCC-3’  5’- CCAACGCTGGGCAATTCTTC-3’ | 70 |
| *POLG* | Forward  Reverse | 5’- GCCTTGCAGATCACCAACCT-3’  5’- TGGGGCAAGTCATTCAGACC-3’ | 71 |


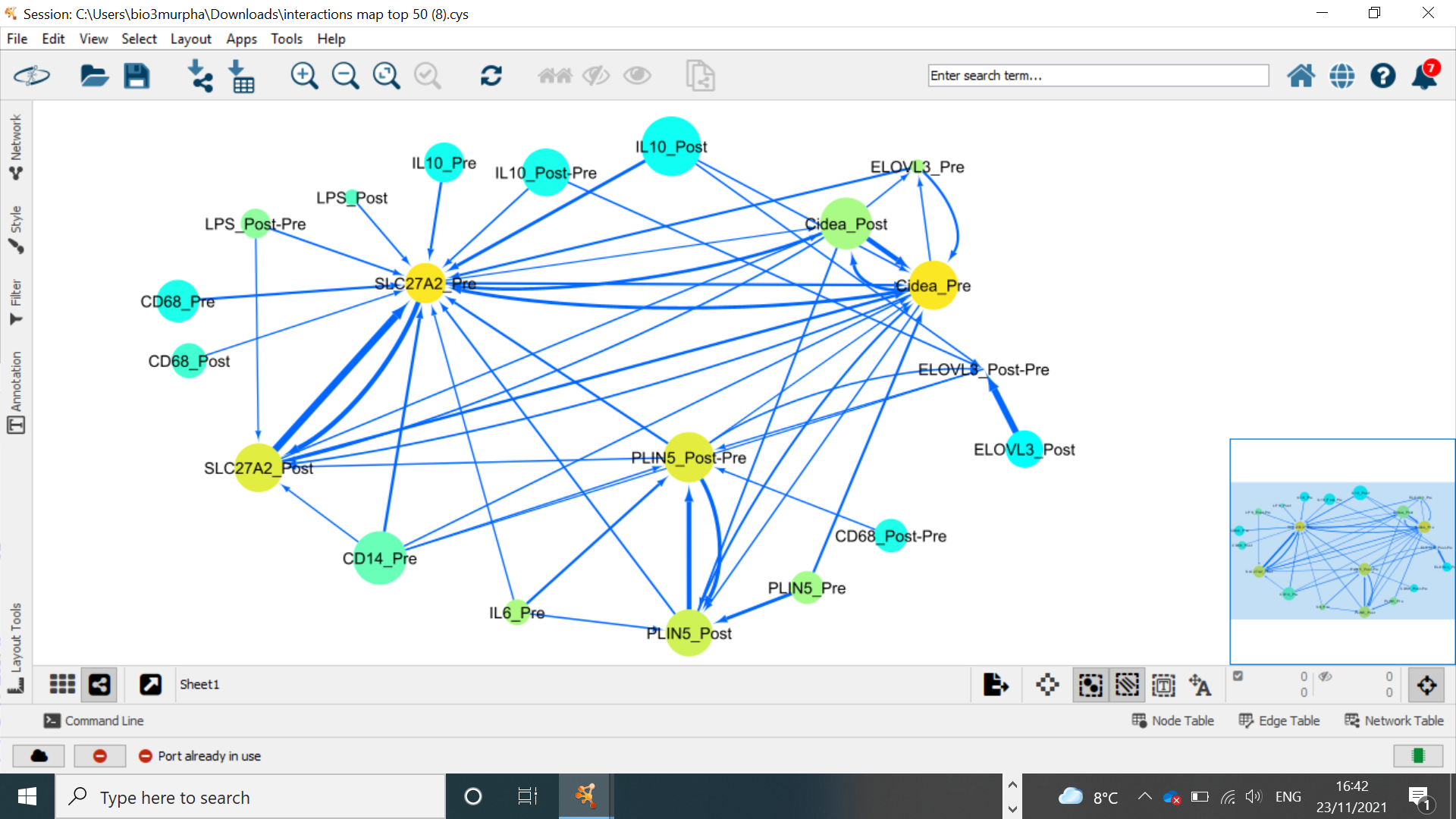


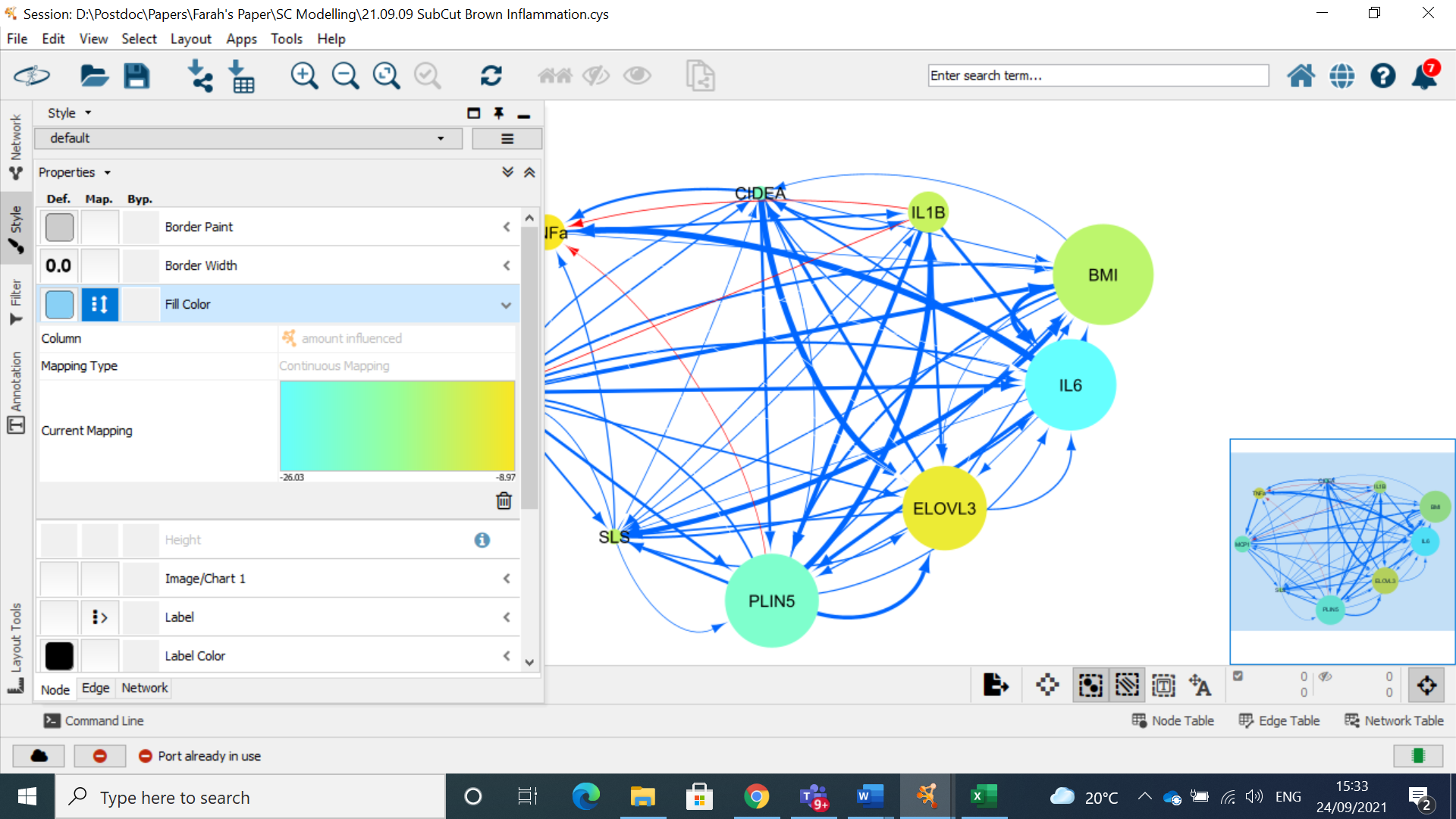


Most influenced

Least influenced

Most influential

Least influential

**Figure S1: Artificial Neural Network Inference of Brown and Inflammatory Genes Pre- and Post-Bariatric Surgery.** Gene expression of brown genes: Cidea (cell death activator CIDE-A), ELOVL3 (elongation of very long chain fatty acids protein 3), PLIN5 (perilipin 5) and SLC27A2 (solute carrier family 27 member 2); and inflammatory genes: IL10 (interleukin 10), CD68 (cluster of differentiation 68), IL6 (interleukin 6), CD14 (cluster of differentiation 14), along with serum endotoxin (LPS) concentration were measured pre- and 6 months post-bariatric surgery. These values, as well as the change between pre- and post-surgery values, were input into an artificial neural network showcasing the interactions between them. Blue arrows indicate a negative regulation, size of the arrow indicates the amount of regulation, size of the node indicates the level of influence on that gene.

**mRNA UCP1**


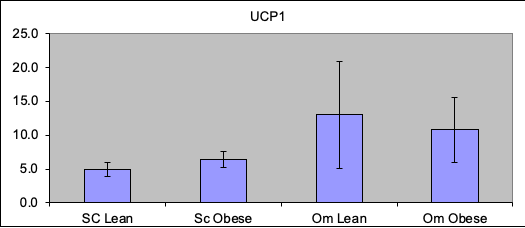


**Sc Lean**

**Sc Obese**

**Om Lean**

**Om Obese**

**Figure S2: UCP1 Expression in Lean and Obese Subcutaneous and Omental Adipose Tissue.** The expression of Uncoupling Protein 1 (UCP1) was measured in subcutaneous (Sc) and omental (Om) adipose tissue depots via RT-PCR using L19 as a housekeeping control. No significant difference was seen between Sc and Om depots.


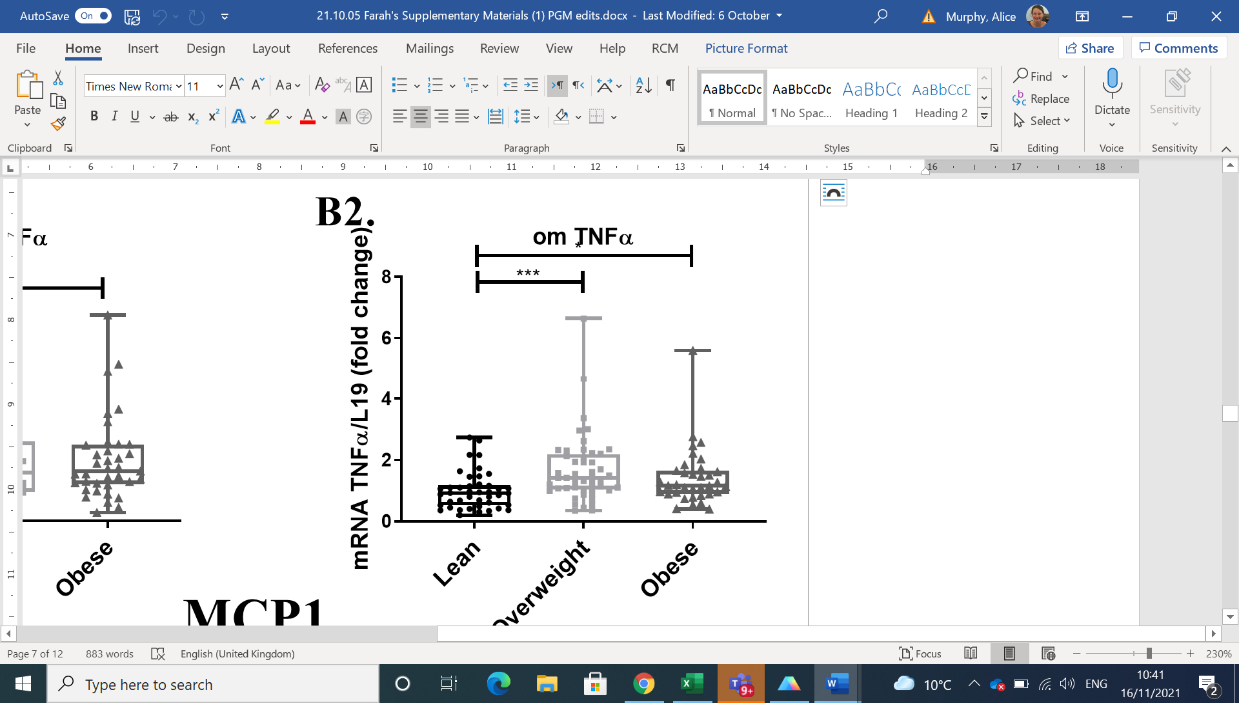


**Figure S3: Inflammatory Markers are Increased with BMI.** Key inflammatory markers interleukin 6 (IL6), tumour necrosis factor alpha (TNFα), monocyte chemotactic protein-1 (MCP1) and interleukin 1 beta (IL1β) were measured in lean (n=44), overweight (n=49) and obese participants (n=37) using RT-PCR with L19 as a housekeeping control gene. The one-way ANOVA test was used to test significance levels: *p<0.05, **p<0.01, ***p<0.001, ****p<0.0001. Whiskers represent minimum and maximum values.


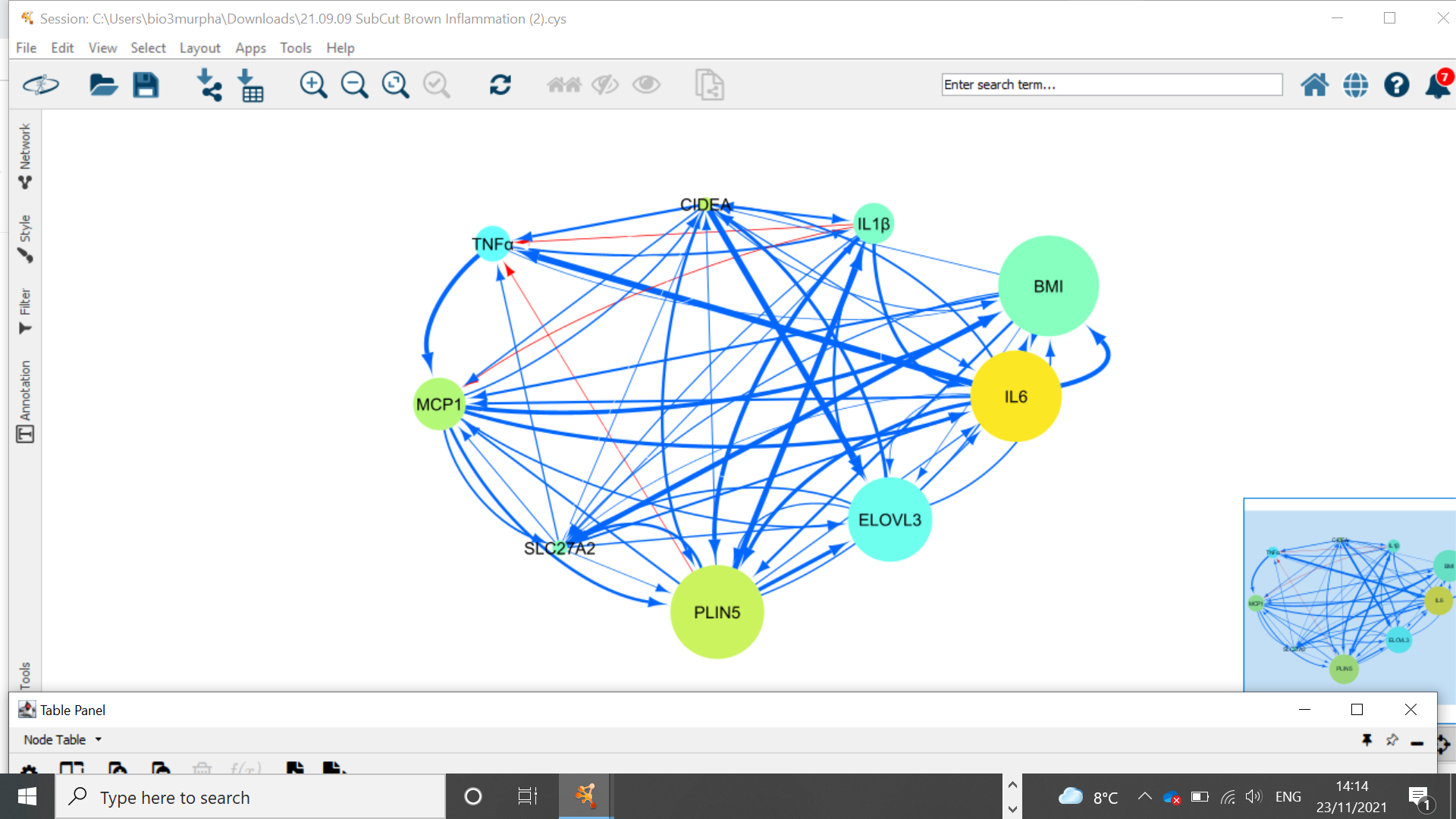


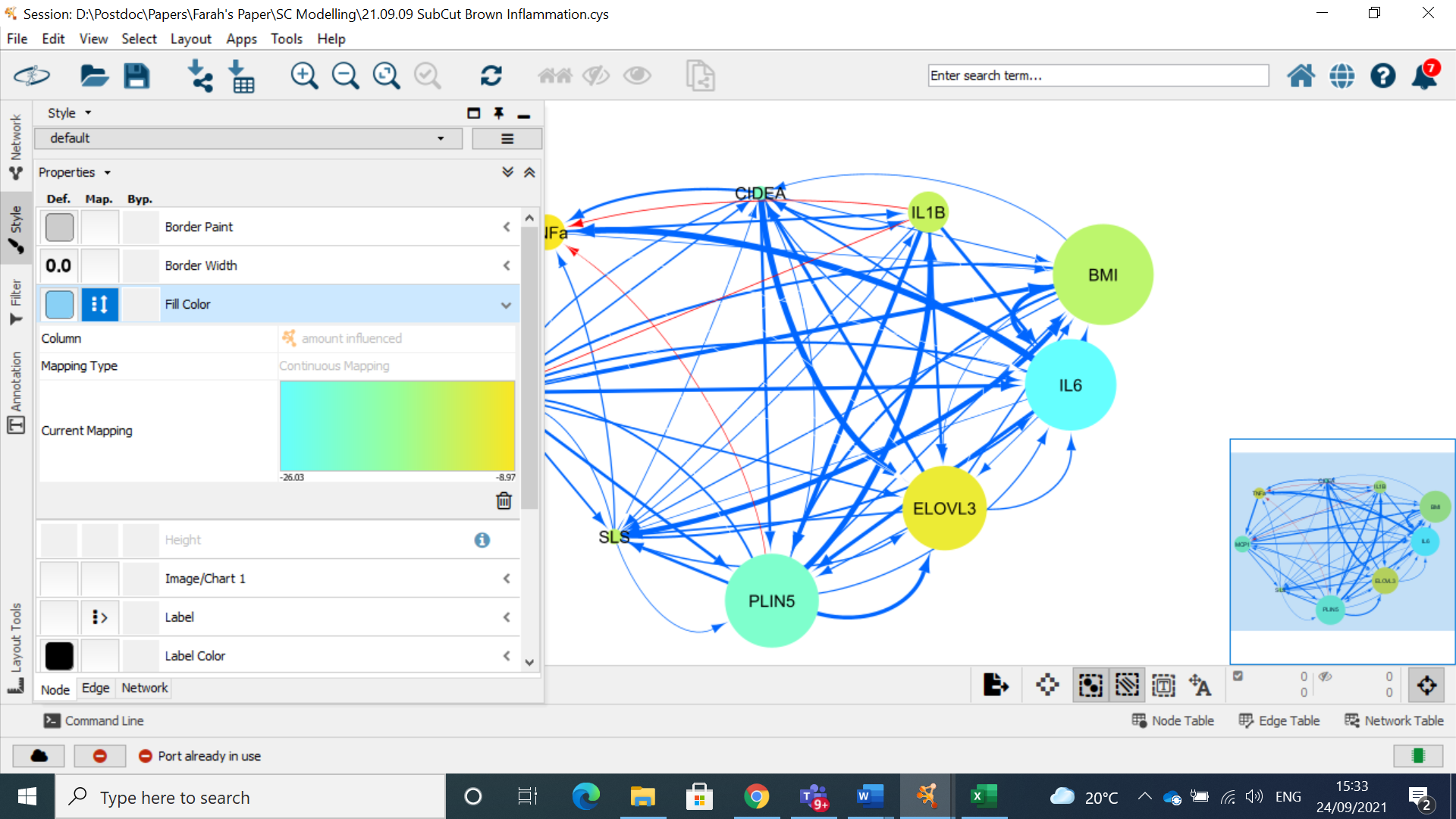


Least influenced

Most influenced

Most influential

Least influential

**Figure S4: Artificial Neural Network Inference of Brown and Inflammatory Genes in Subcutaneous Adipose Tissue.** Gene expression of brown genes: CIDEA (cell death activator CIDE-A), ELOVL3 (elongation of very long chain fatty acids protein 3), PLIN5 (perilipin 5) and SLC27A2 (solute carrier family 27 member 2); and inflammatory genes: IL6 (interleukin-6), MCP1 (monocyte chemotactic protein-1), TNFα (tumour necrosis factor-alpha), and IL1β (interleukin-1beta) were measured in subcutaneous adipose tissue. These values were input into an artificial neural network, along with BMI, showcasing the interactions between them. Blue arrows indicate a negative regulation, red arrows indicate positive regulation, size of the arrow indicates the amount of regulation, size of the node indicates the overall level of influence that gene has, node colour indicates the level of influence that gene experiences.


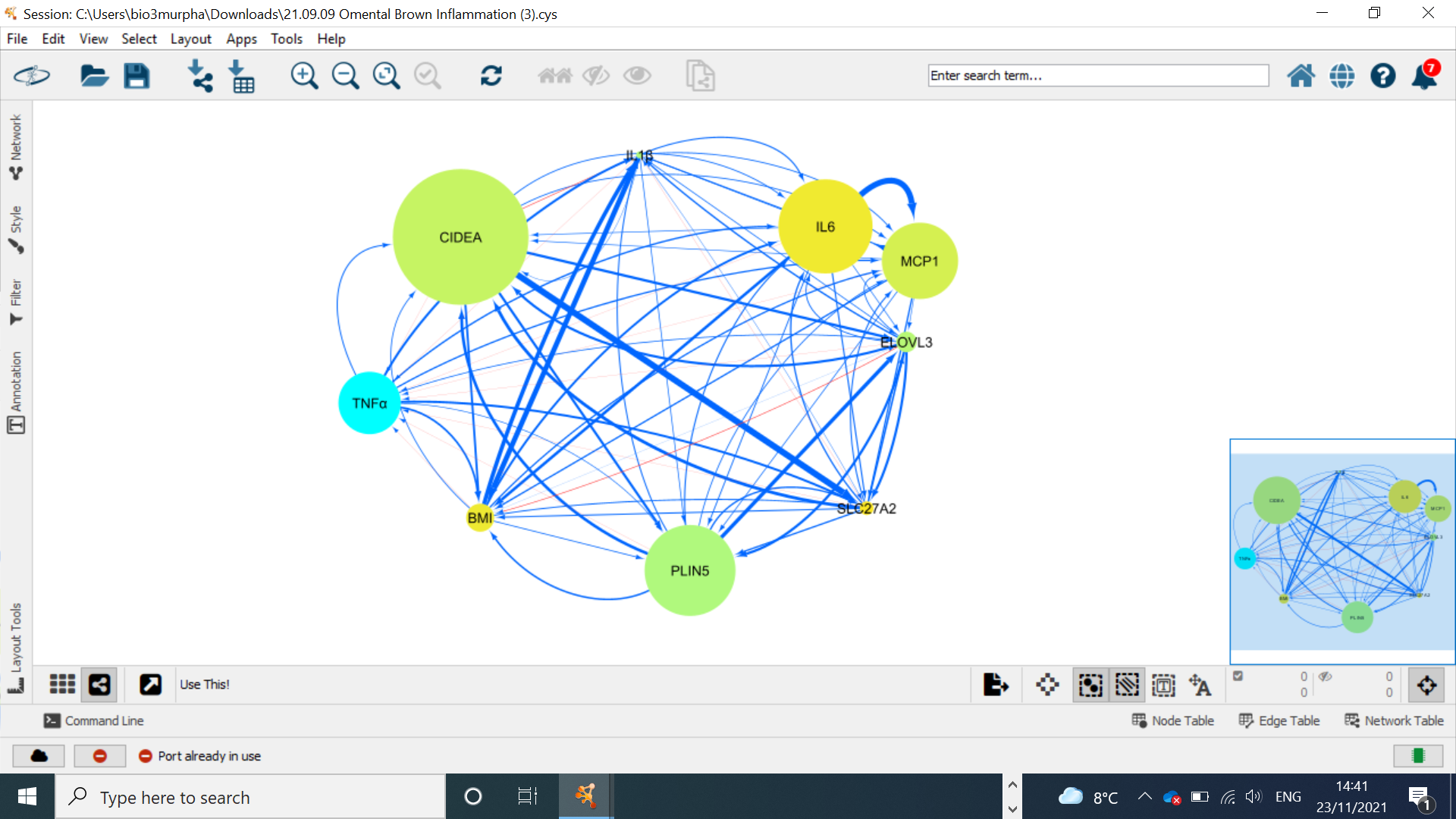


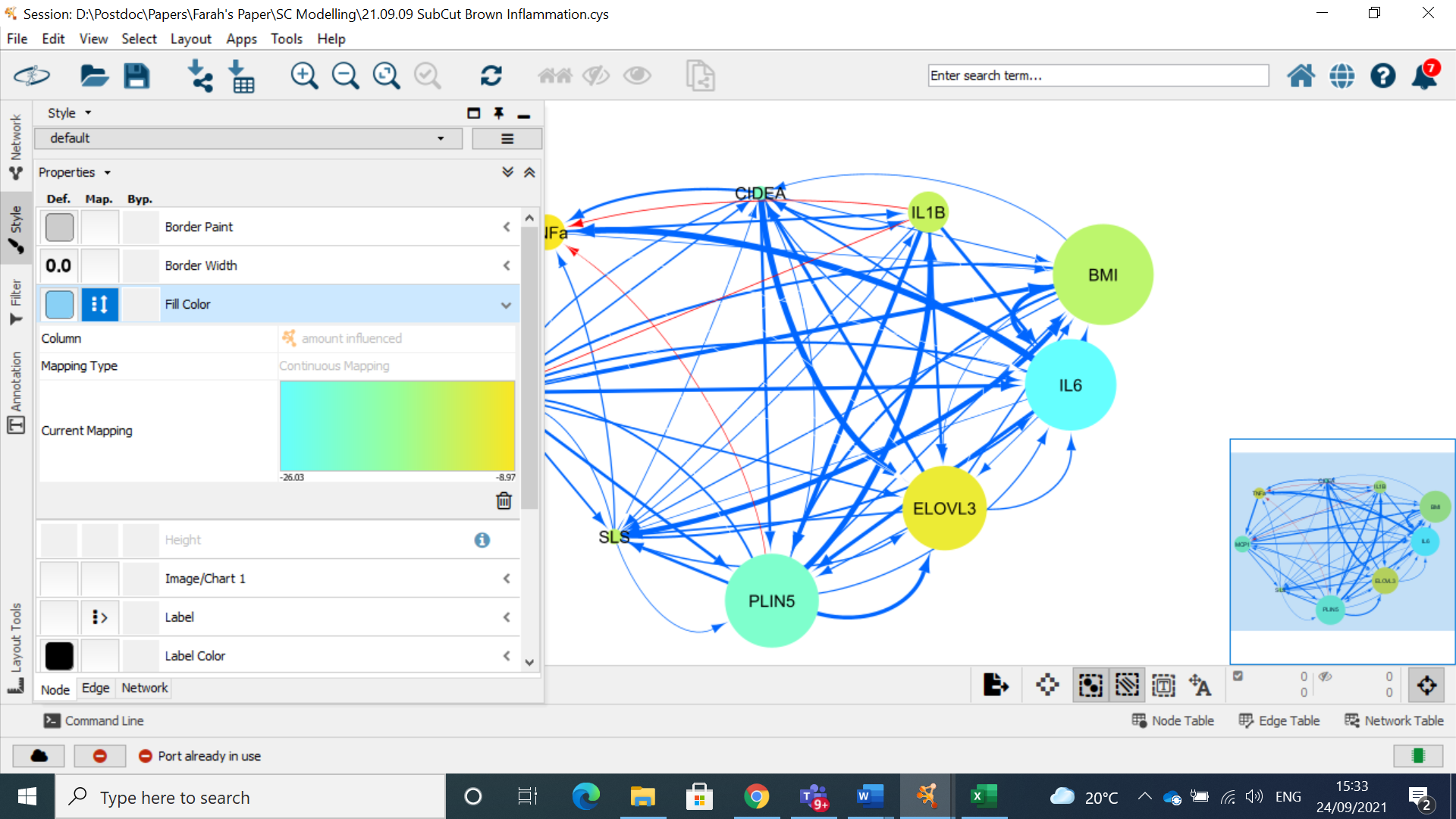


Most influenced

Least influenced

Most influential

Least influential

**Figure S5: Artificial Neural Network Inference of Brown and Inflammatory Genes in Omental Adipose Tissue.** Gene expression of brown genes: Cidea (cell death activator CIDE-A), ELOVL3 (elongation of very long chain fatty acids protein 3), PLIN5 (perilipin 5) and SLC27A2 (solute carrier family 27 member 2); and inflammatory genes: IL6 (interleukin-6), MCP1 (monocyte chemotactic protein-1), TNFα (tumour necrosis factor-alpha), and IL1β (interleukin-1beta) measured in omental adipose tissue. These values were input into an artificial neural network, along with BMI, showcasing the interactions between them. Blue arrows indicate a negative regulation, red arrows indicate positive regulation, size of the arrow indicates the amount of regulation, size of the node indicates the overall level of influence that gene has, node colour indicates the level of influence that gene experiences.

**Figure S6: AP2 Gene Expression During Differentiation with LPS Treatment.** Lean and obese primary human adipocytes were cultured and differentiated with or without 100ng/ml Lipopolysaccharide (LPS), 2µM Rosiglitazone (Rosi), or a combination of the two. When fully differentiated, cells were treated with or without 10µM Isoproterenol (Iso). Gene expression of adipocyte protein 2 (AP2) was assessed to determine any impact on adipocyte differentiation. No significant difference in expression was observed with any treatment, indicating that differentiation of lean and obese adipocytes was not impacted.

 **Figure S7: Effect of LPS on Inflammation in Primary Human Adipocytes.** Lean (**A1, B1**) and obese (**A1, B2**) primary human adipocyte cells were cultured and differentiated with or without 2µM rosiglitazone (Rosi), 100ng/ml Lipopolysaccharide (LPS100), or a combination of the two. Inflammatory genes interleukin 6 (IL6) and monocyte chemotactic protein-1 (MCP1) were measured via RT-PCR using L19 as a housekeeping control. Data represent mean ± SEM. The two-way ANOVA test was used to test significance; *p<0.05, **p<0.01, ***p<0.001, ****p<0.0001 compared to control, † p<0.05, †† p<0.01, ††† p<0.001, †††† p<0.0001 compared to Rosi, x p<0.05, xx p<0.01, xxx p<0.001, xxxx p<0.0001 compared to LPS.


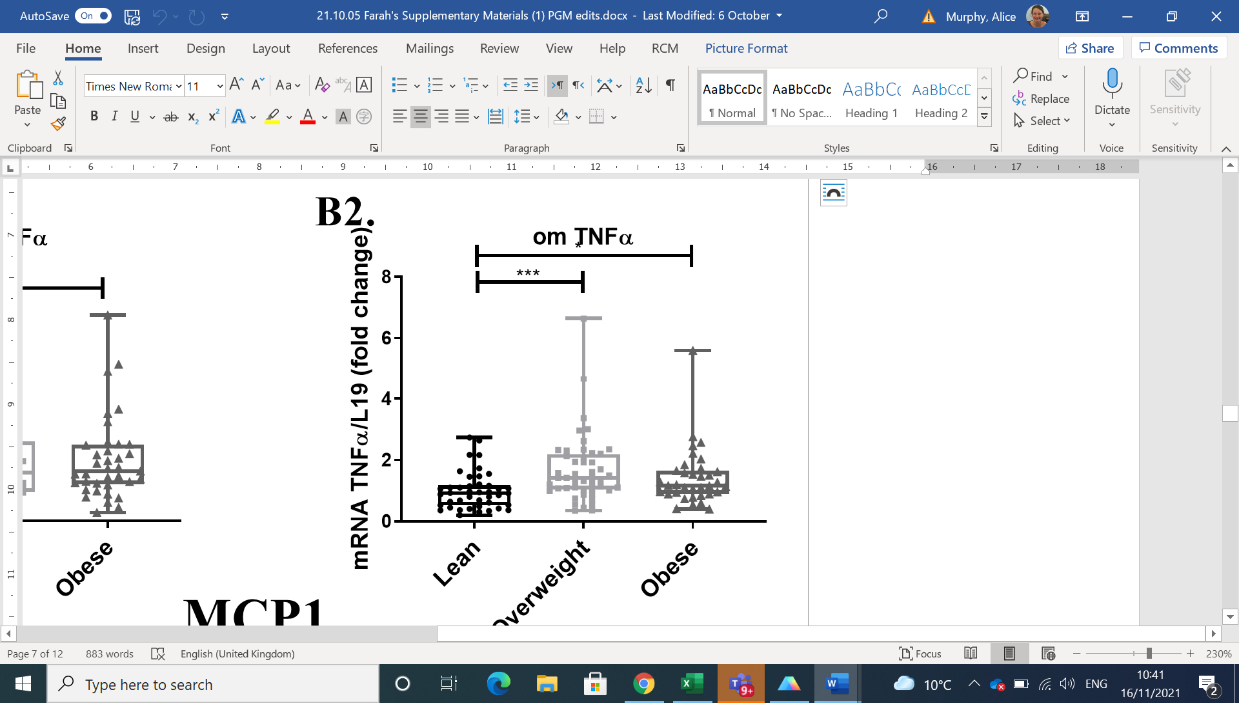

Supplement: Supplementary file 1 — Additional file 1: Table S1. Primer sequences. Sequences of primers used during qRT-PCR. Figure S1. Artificial neural network inference of brown and inflammatory genes pre- and post-bariatric surgery. A network map showing the strength and direction of interactions between genes measured before and after bariatric surgery. Figure S2. UCP1 expression in lean and obese subcutaneous and omental adipose tissue. Gene expression of UCP1 in subcutaneous and omental adipose tissue from lean participants and participants with obesity. Figure S3. Inflammatory markers are increased with BMI. Bar graphs showing the expression of inflammatory genes across participants divided into lean, overweight and obese cohorts. Figure S4. Artificial neural network inference of brown and inflammatory genes in subcutaneous adipose tissue. A network map showing the strength and direction of interactions between genes in subcutaneous adipose tissue. Figure S5. Artificial neural network inference of brown and inflammatory genes in omental adipose tissue. A network map showing the strength and direction of interactions between genes in omental adipose tissue. Figure S6. AP2 gene expression during differentiation with LPS treatment. Gene expression of AP2 demonstrating that differentiation of adipocytes is not affected by cellular treatments. Figure S7. Effect of LPS on inflammation in primary human adipocytes. Expression of inflammatory genes in primary human adipocytes following LPS treatment. [file 12916_2023_2857_MOESM1_ESM.docx]
