# Supplementary figures and images for: The impact of metabolic endotoxaemia on the browning process in human adipocytes
Source: BMC Med. 2023 Apr 19;21:154. doi: 10.1186/s12916-023-02857-z (PMC10116789; doi:10.1186/s12916-023-02857-z)

**UCP1**


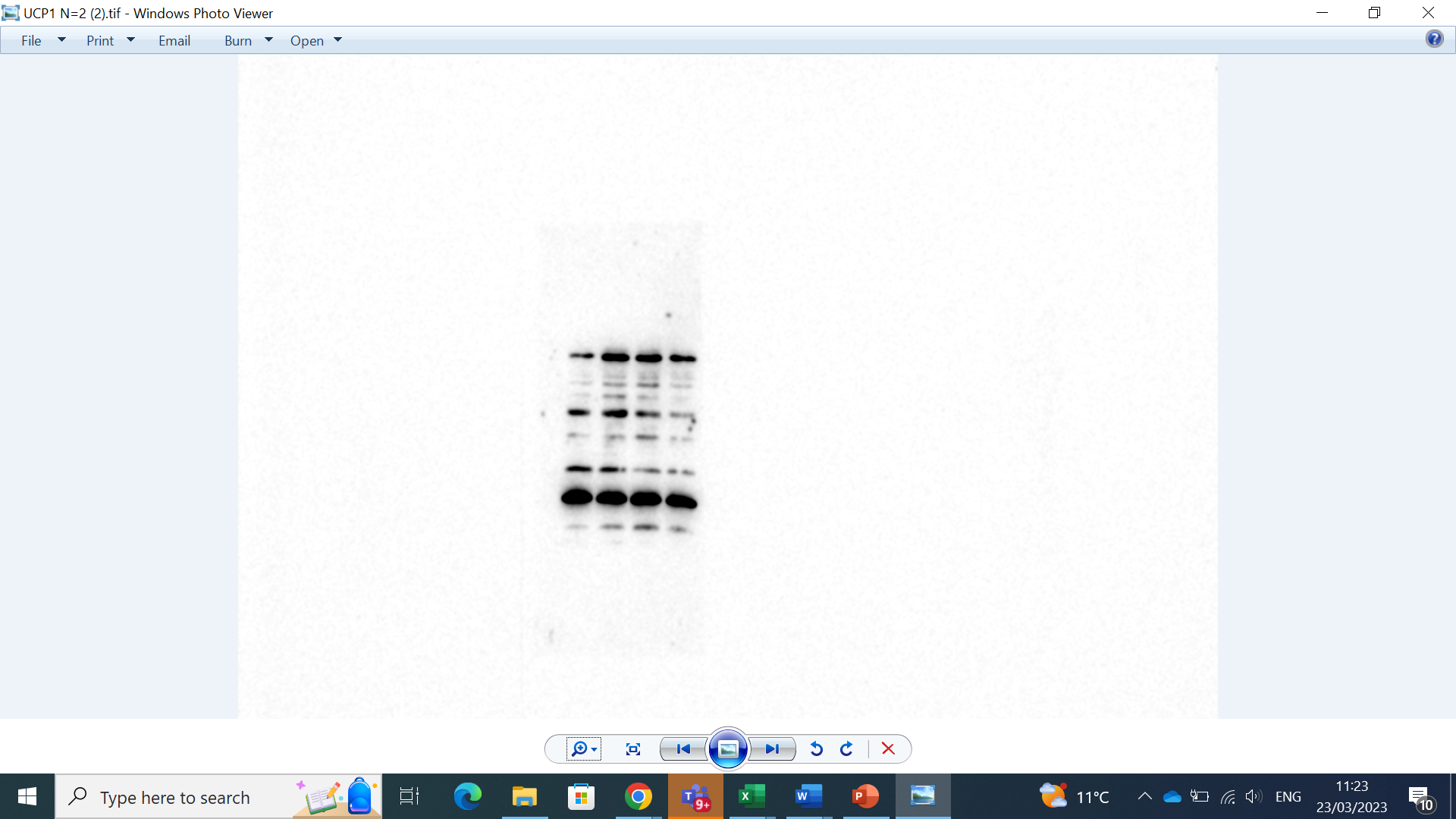


50kDa

37kDa

20kDa


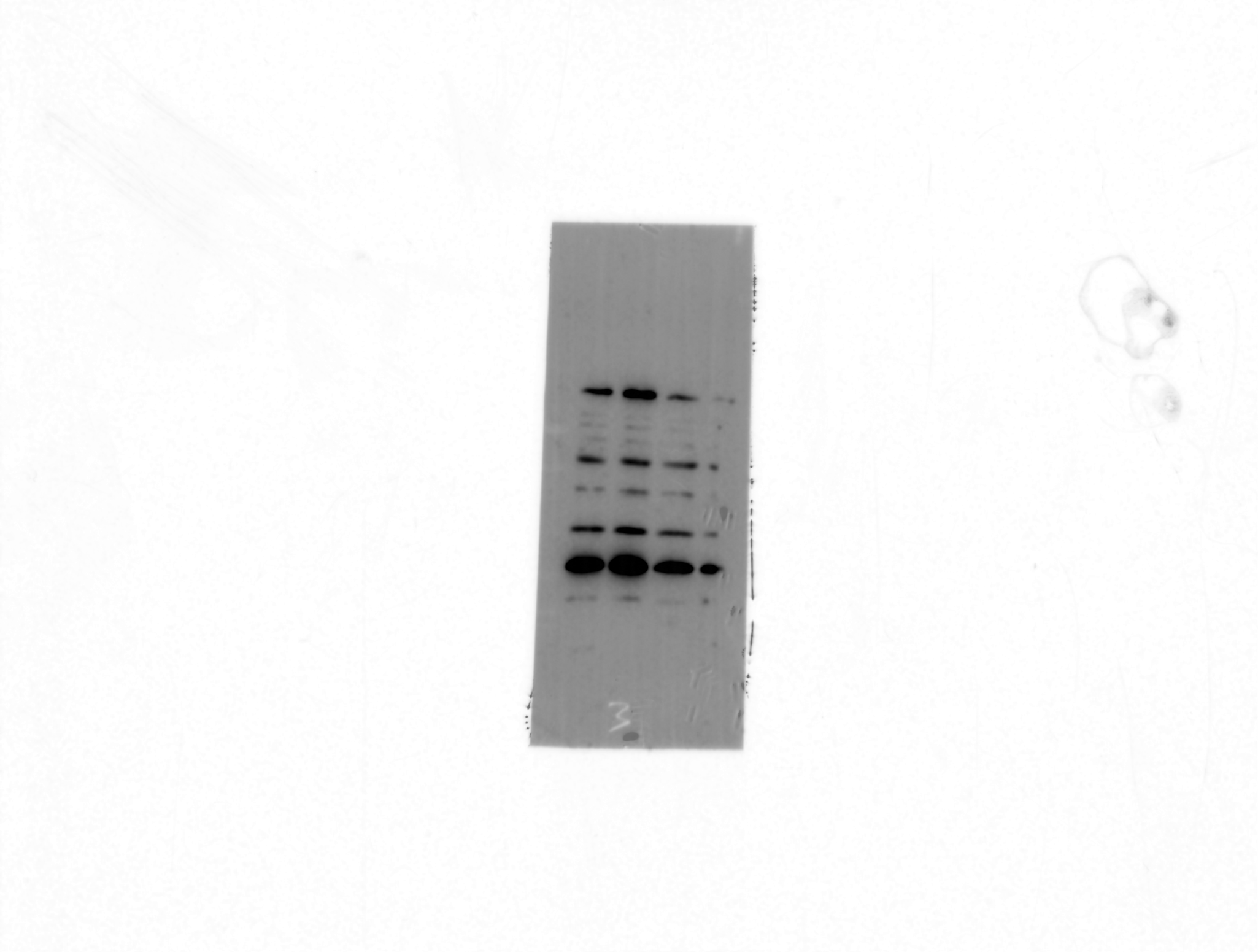


50kDa

37kDa

20kDa


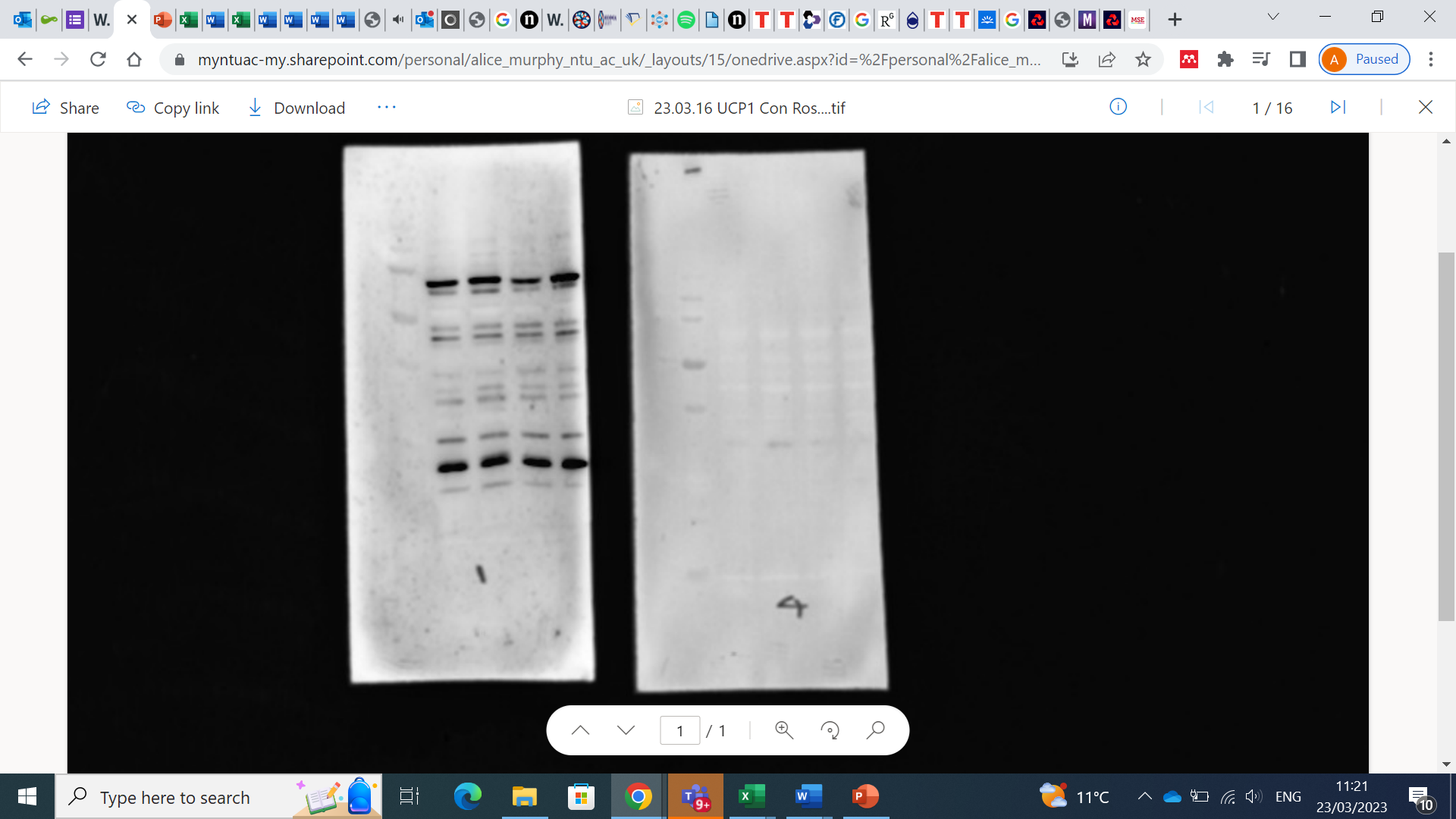


50kDa

37kDa

20kDa

**β-Actin**


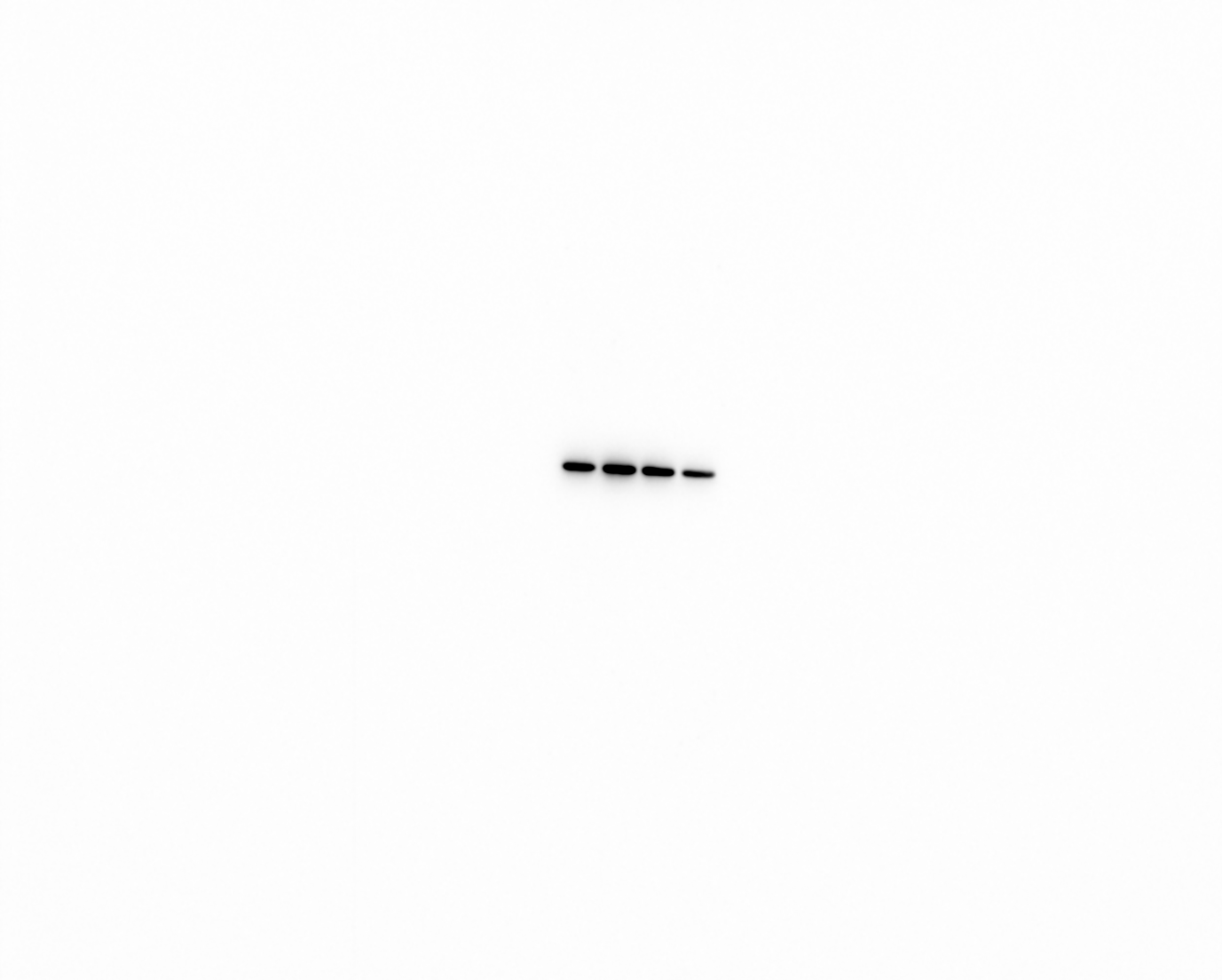

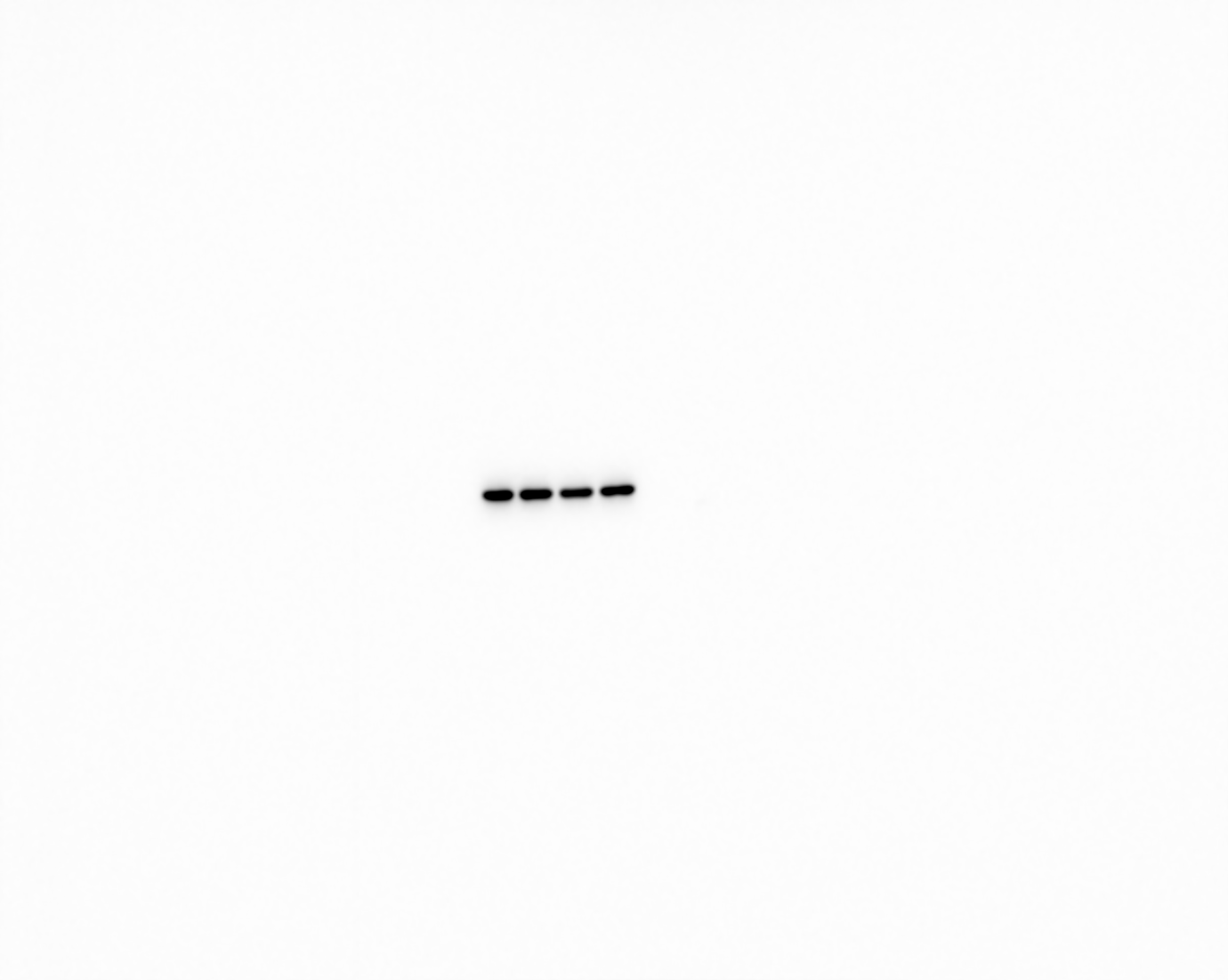

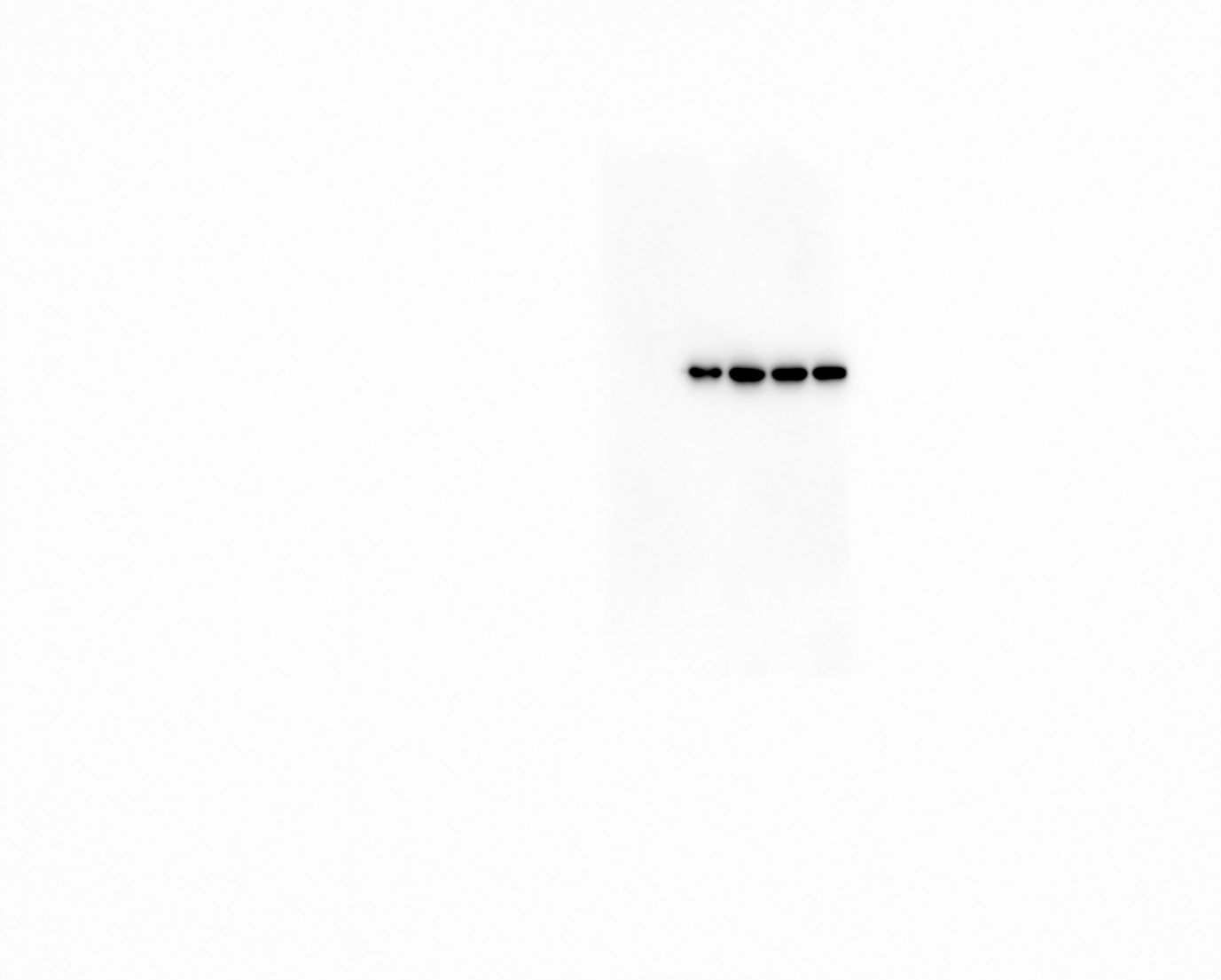

Supplement: Supplementary file 2 — Additional file 2. [file 12916_2023_2857_MOESM2_ESM.docx]
